# Supplementary figures and images for: Loss of Myo19 increases metastasis by enhancing microenvironmental ROS gradient and chemotaxis
Source: EMBO Rep. 2024 Jan 26;25(3):9. doi: 10.1038/s44319-023-00052-y (PMC10933354; doi:10.1038/s44319-023-00052-y)

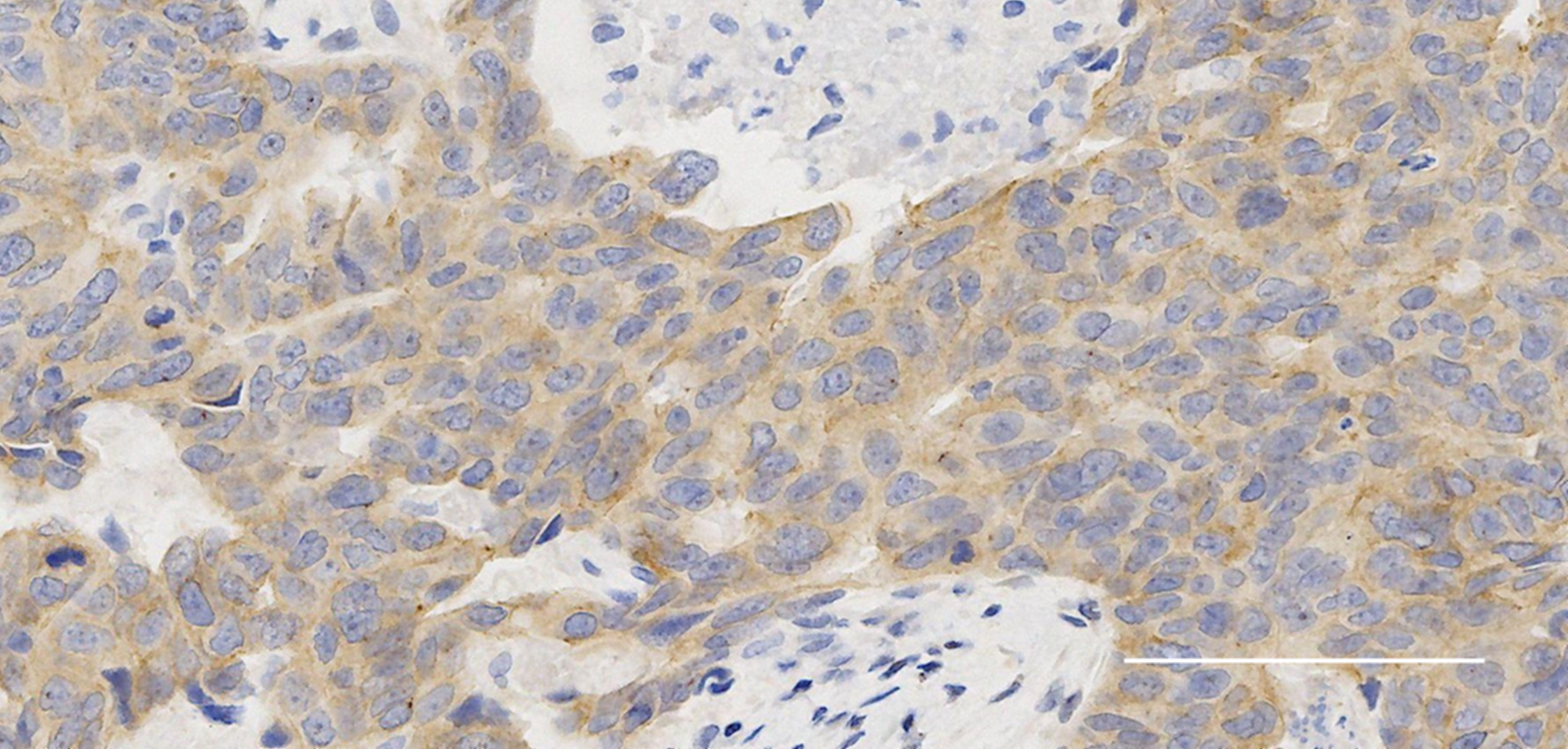

Supplement: Supplementary file 2 — Source Data Fig. 1 [file 44319_2023_52_MOESM2_ESM.zip › Figure 1/1A/Metastasis.tif]

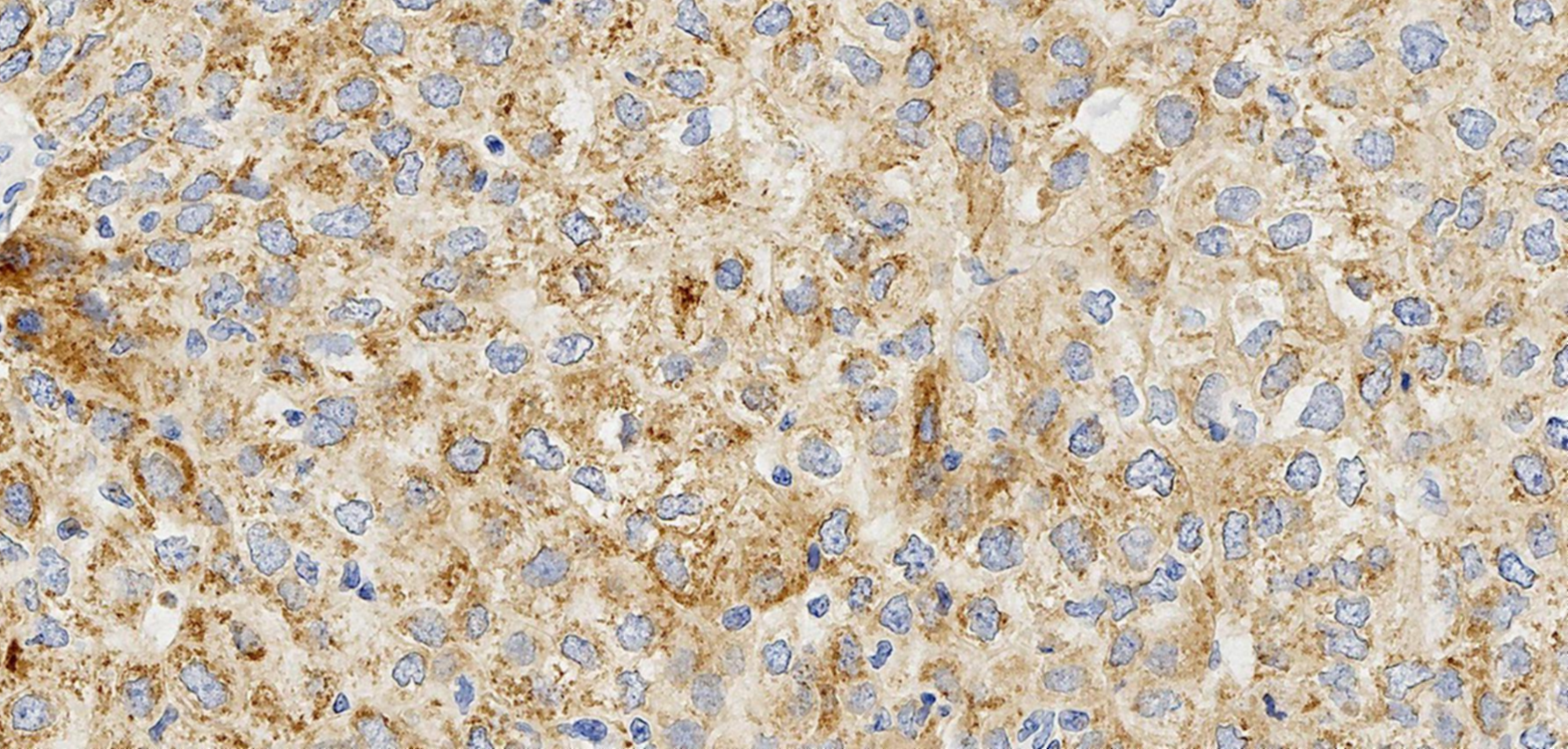

Supplement: Supplementary file 2 — Source Data Fig. 1 [file 44319_2023_52_MOESM2_ESM.zip › Figure 1/1A/Non metastasis.tif]

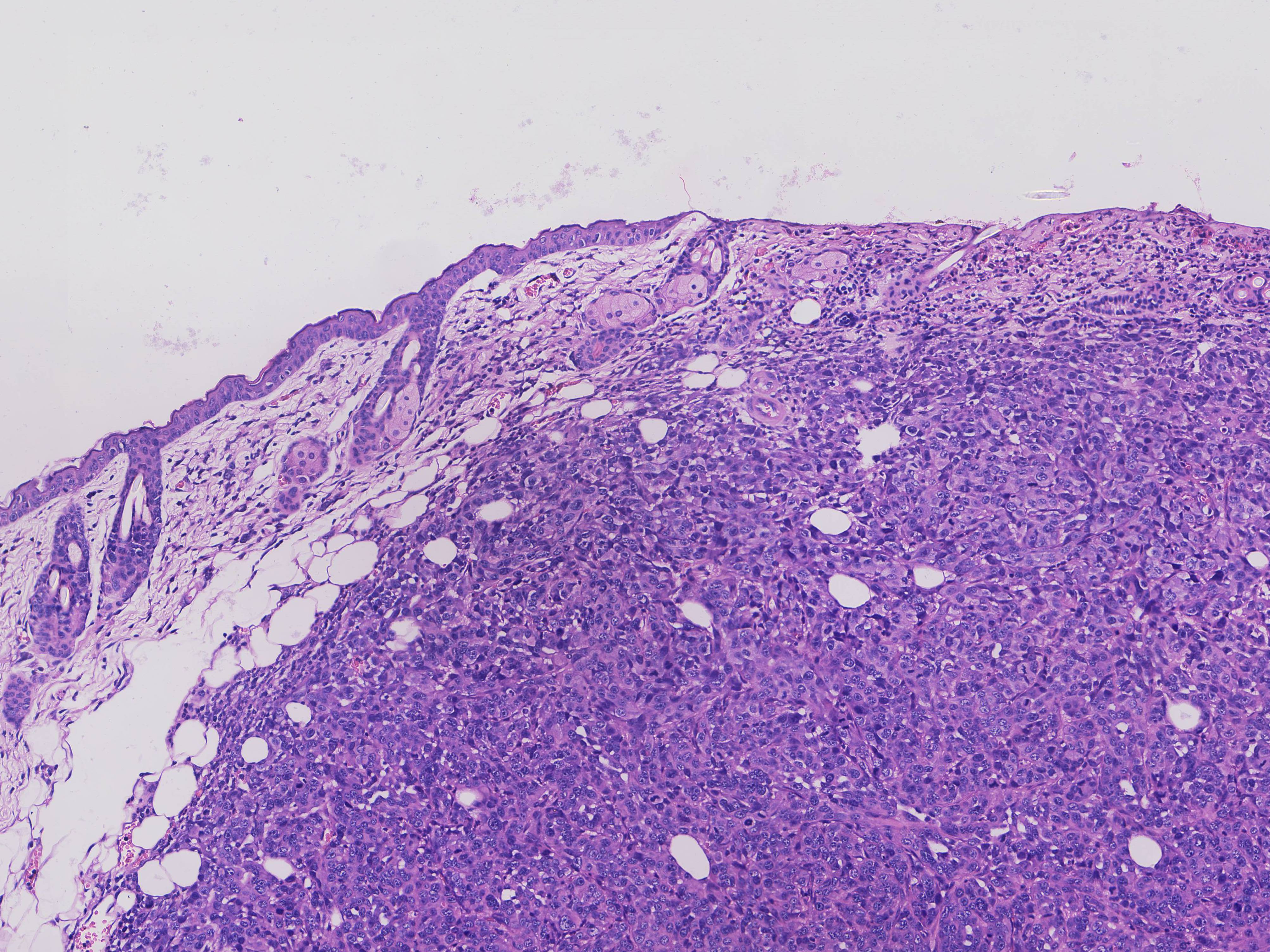

Supplement: Supplementary file 2 — Source Data Fig. 1 [file 44319_2023_52_MOESM2_ESM.zip › Figure 1/1D/Dermis and epidermis-Invasion.tif]

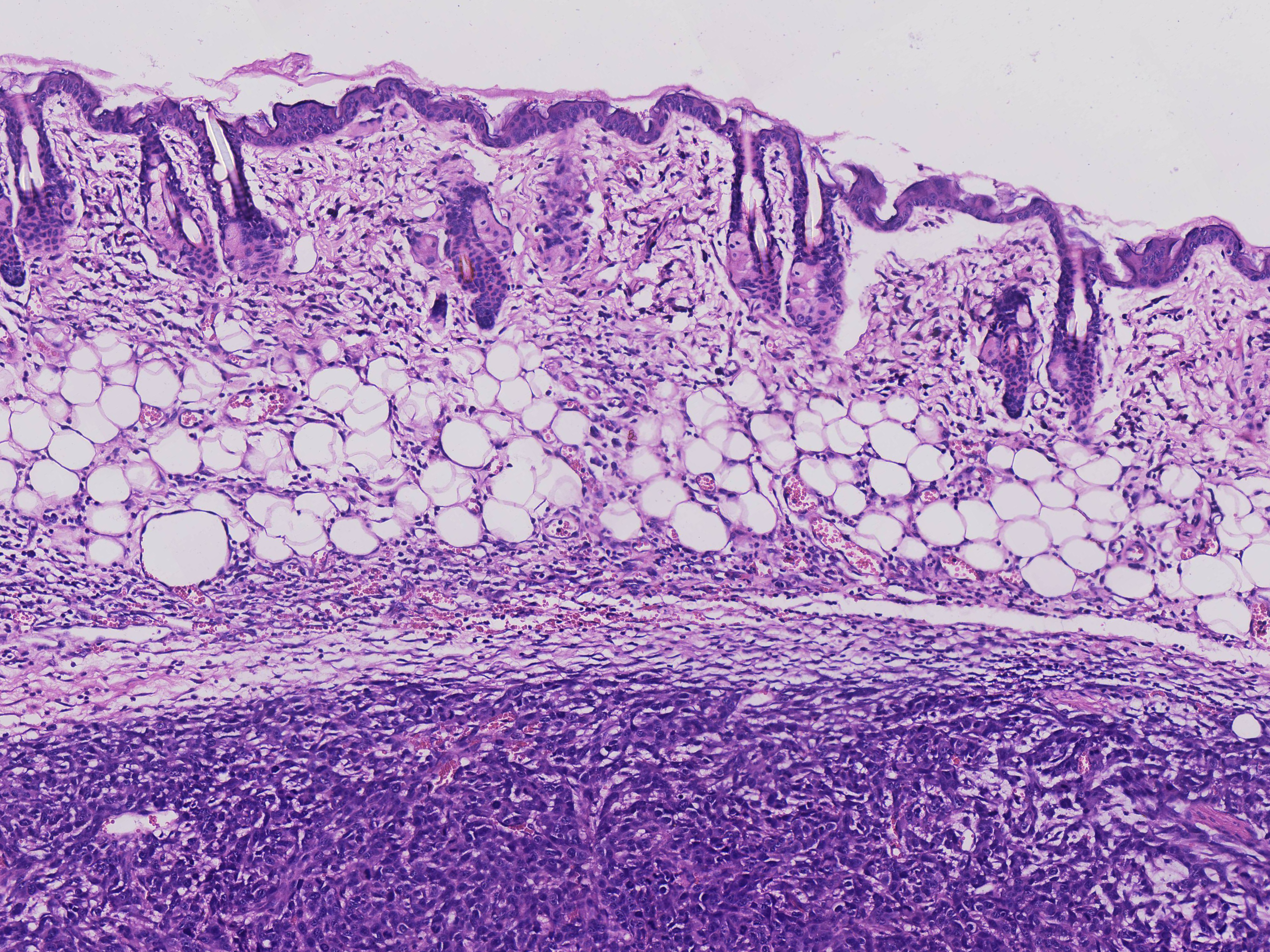

Supplement: Supplementary file 2 — Source Data Fig. 1 [file 44319_2023_52_MOESM2_ESM.zip › Figure 1/1D/Dermis and epidermis-Non invasion.tif]

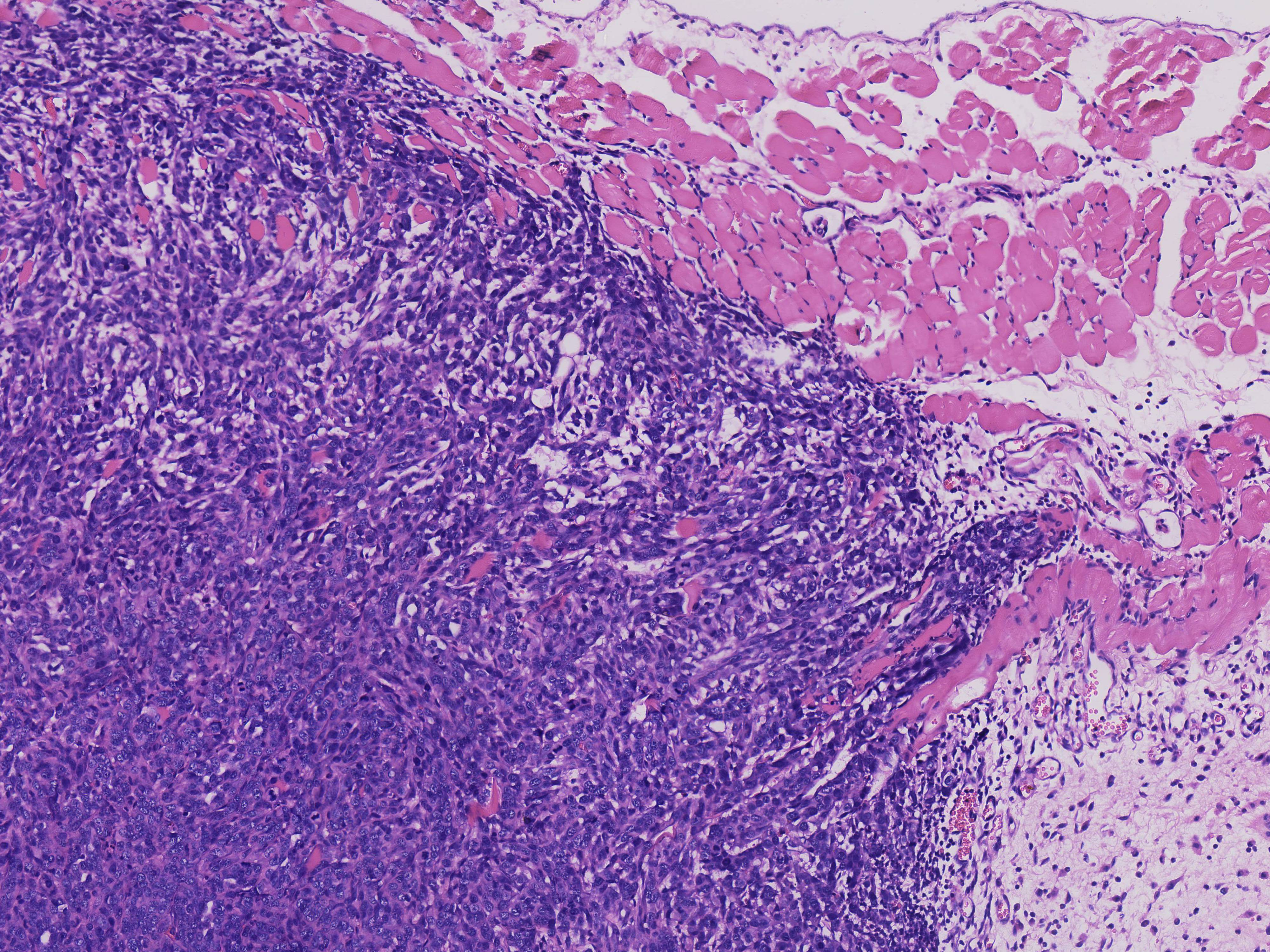

Supplement: Supplementary file 2 — Source Data Fig. 1 [file 44319_2023_52_MOESM2_ESM.zip › Figure 1/1D/Muscle-Invasion.tif]

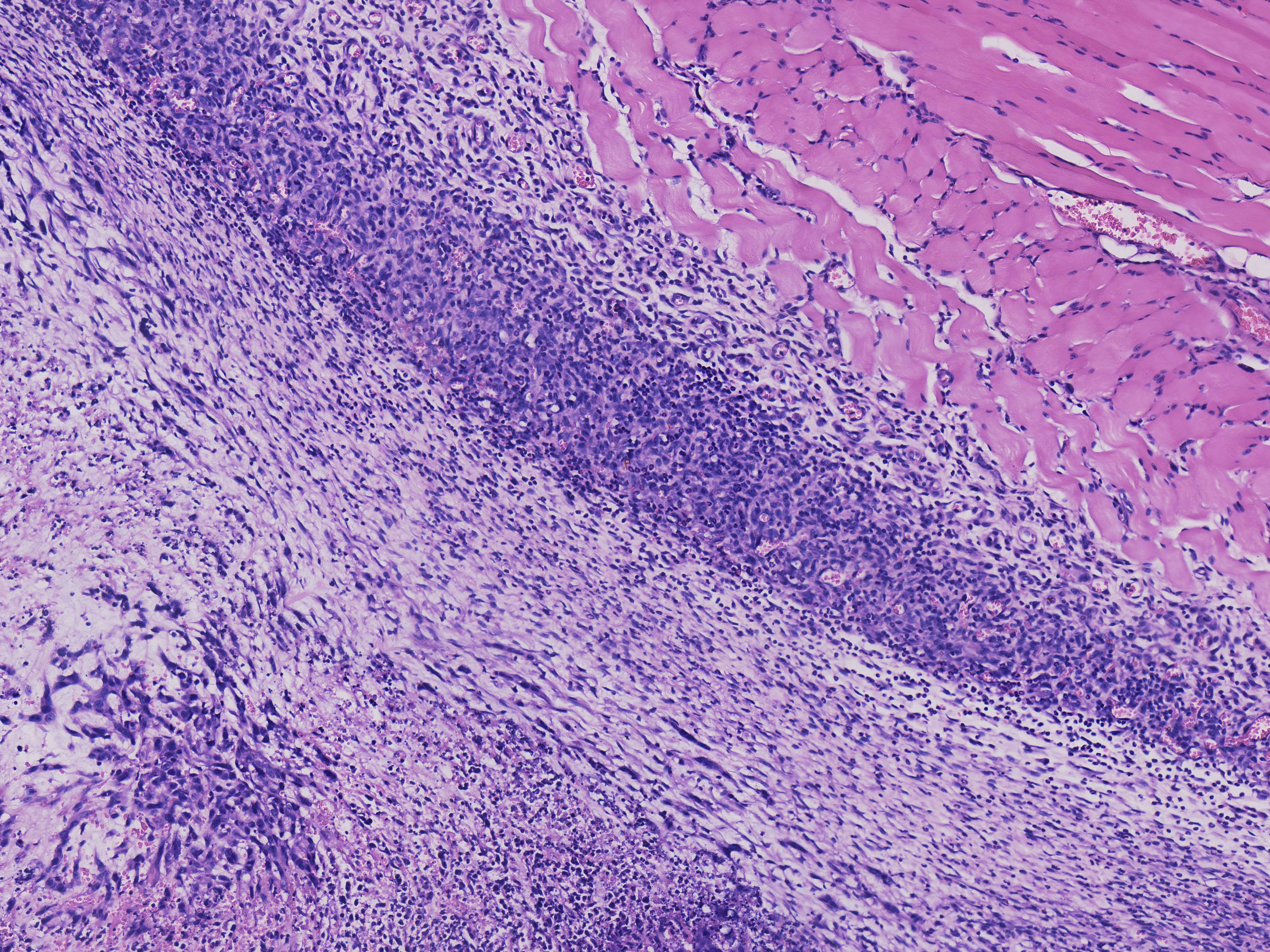

Supplement: Supplementary file 2 — Source Data Fig. 1 [file 44319_2023_52_MOESM2_ESM.zip › Figure 1/1D/Muscle-Non invasion.tif]

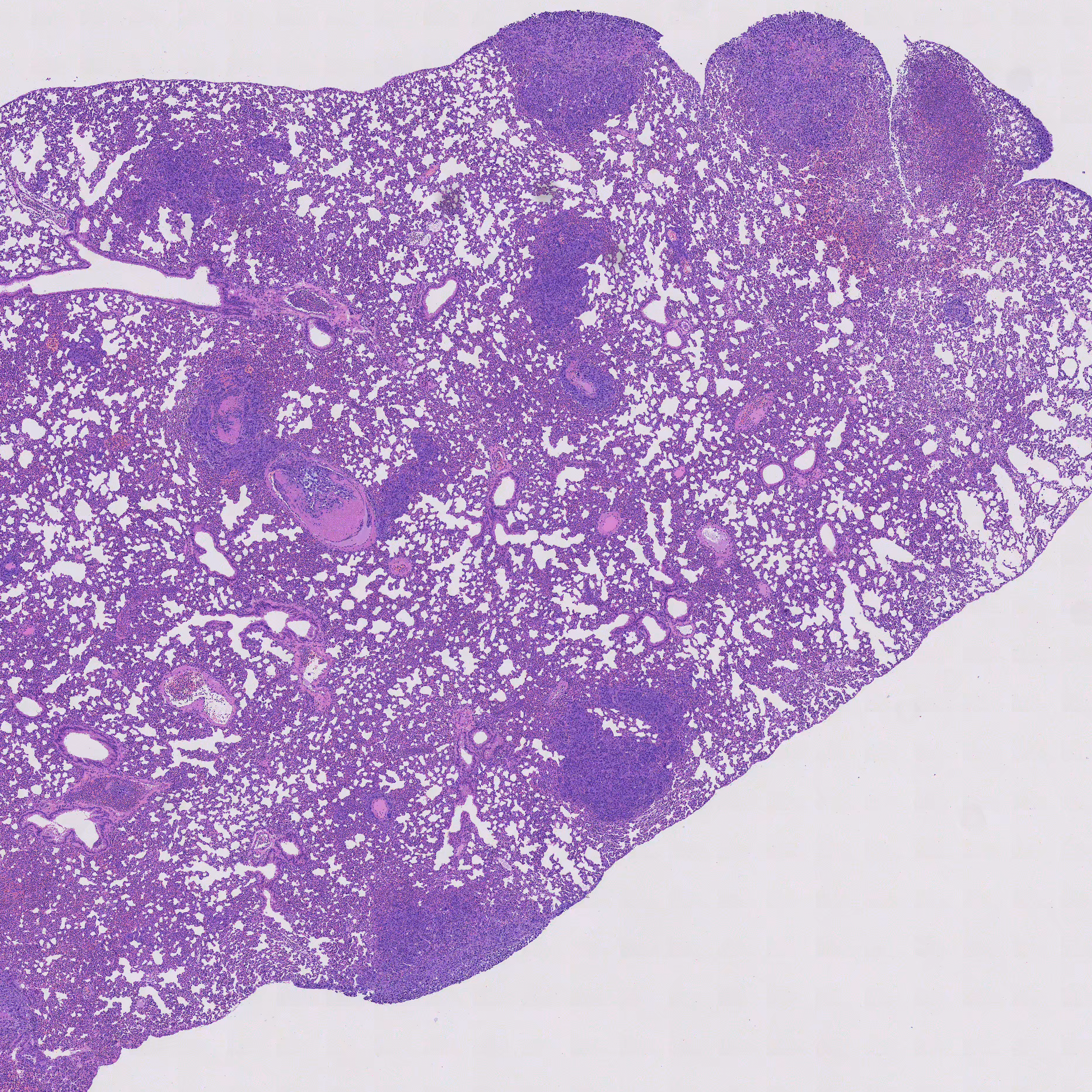

Supplement: Supplementary file 2 — Source Data Fig. 1 [file 44319_2023_52_MOESM2_ESM.zip › Figure 1/1G/Mic60 KD.tif]

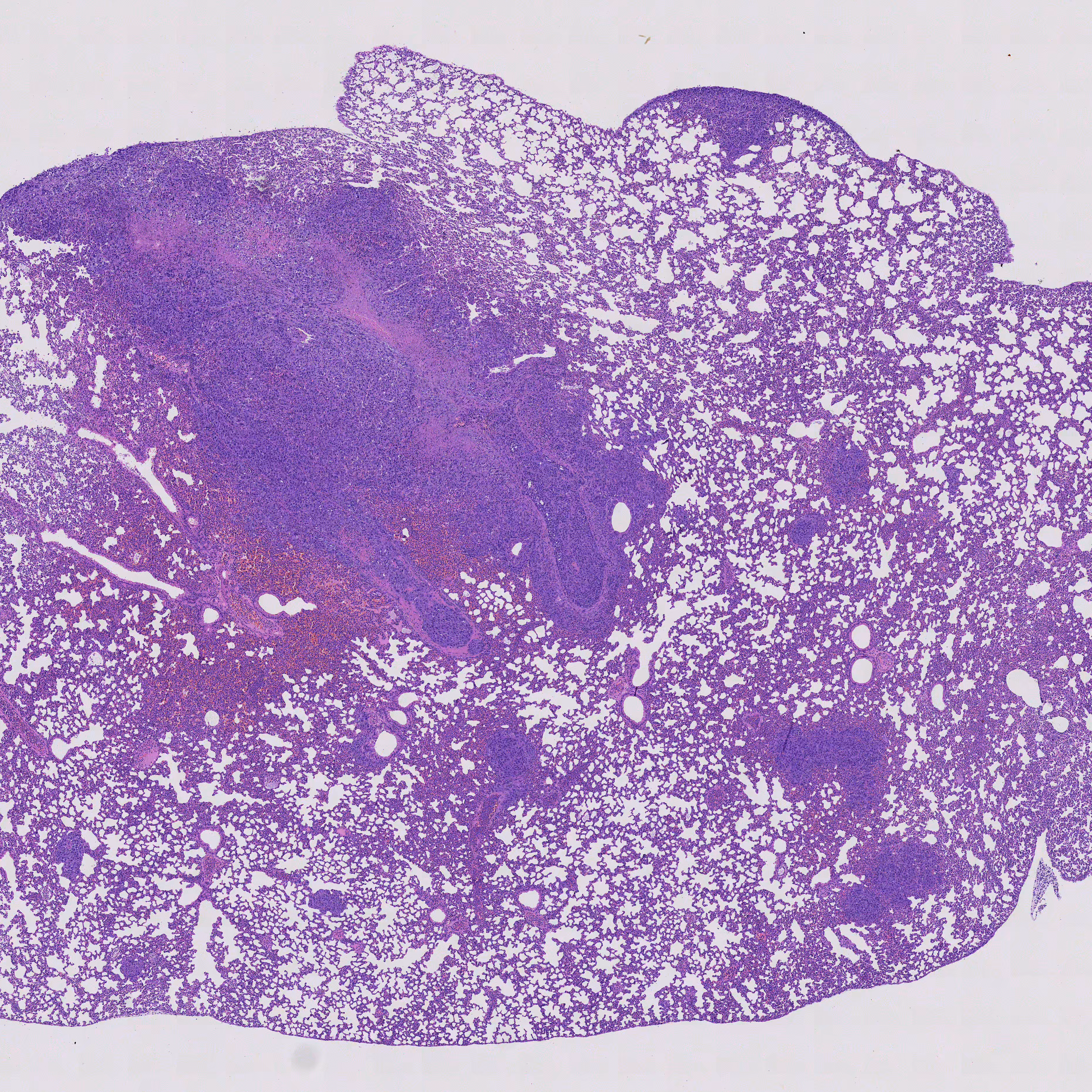

Supplement: Supplementary file 2 — Source Data Fig. 1 [file 44319_2023_52_MOESM2_ESM.zip › Figure 1/1G/Myo19 KD.tif]

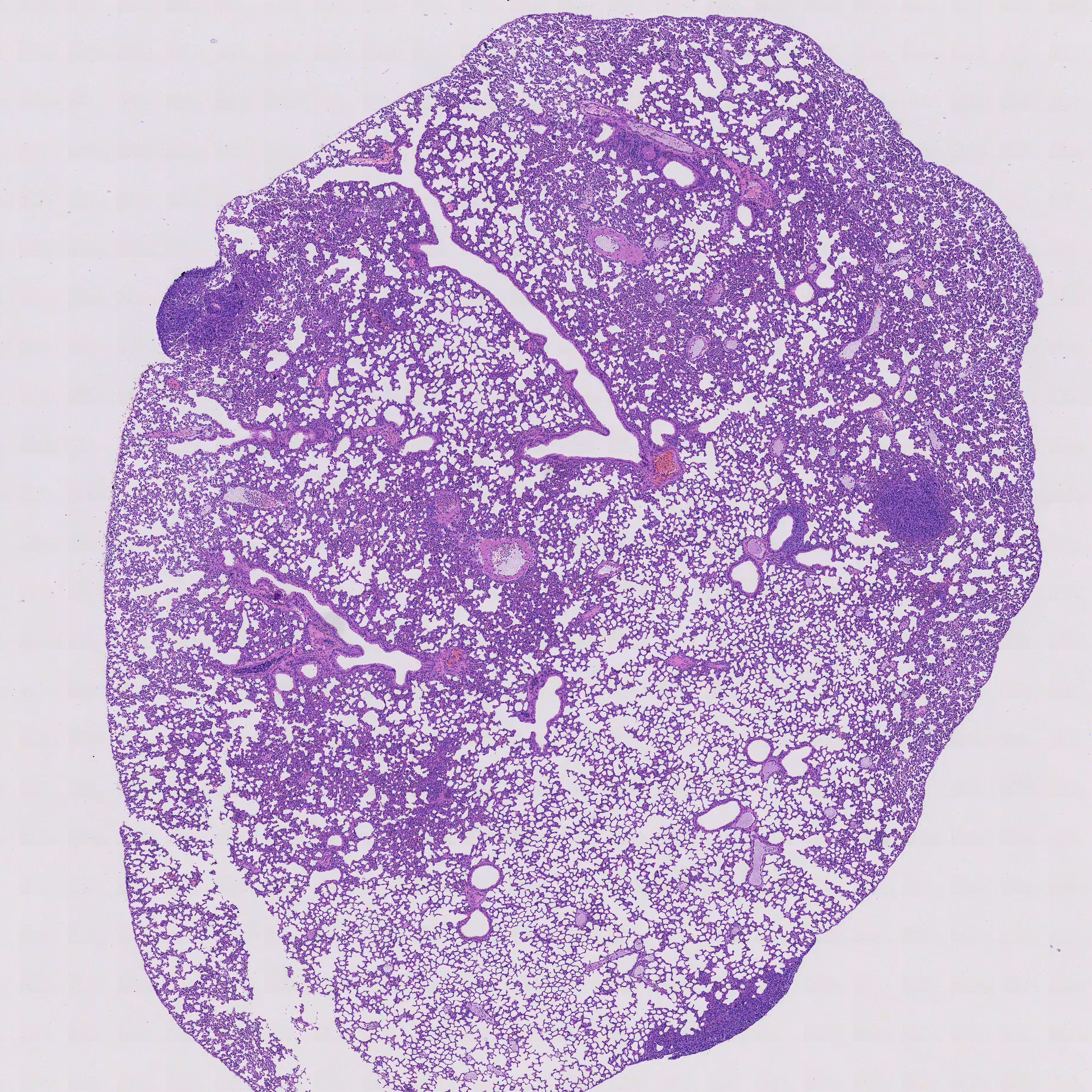

Supplement: Supplementary file 2 — Source Data Fig. 1 [file 44319_2023_52_MOESM2_ESM.zip › Figure 1/1G/scramble.tif]

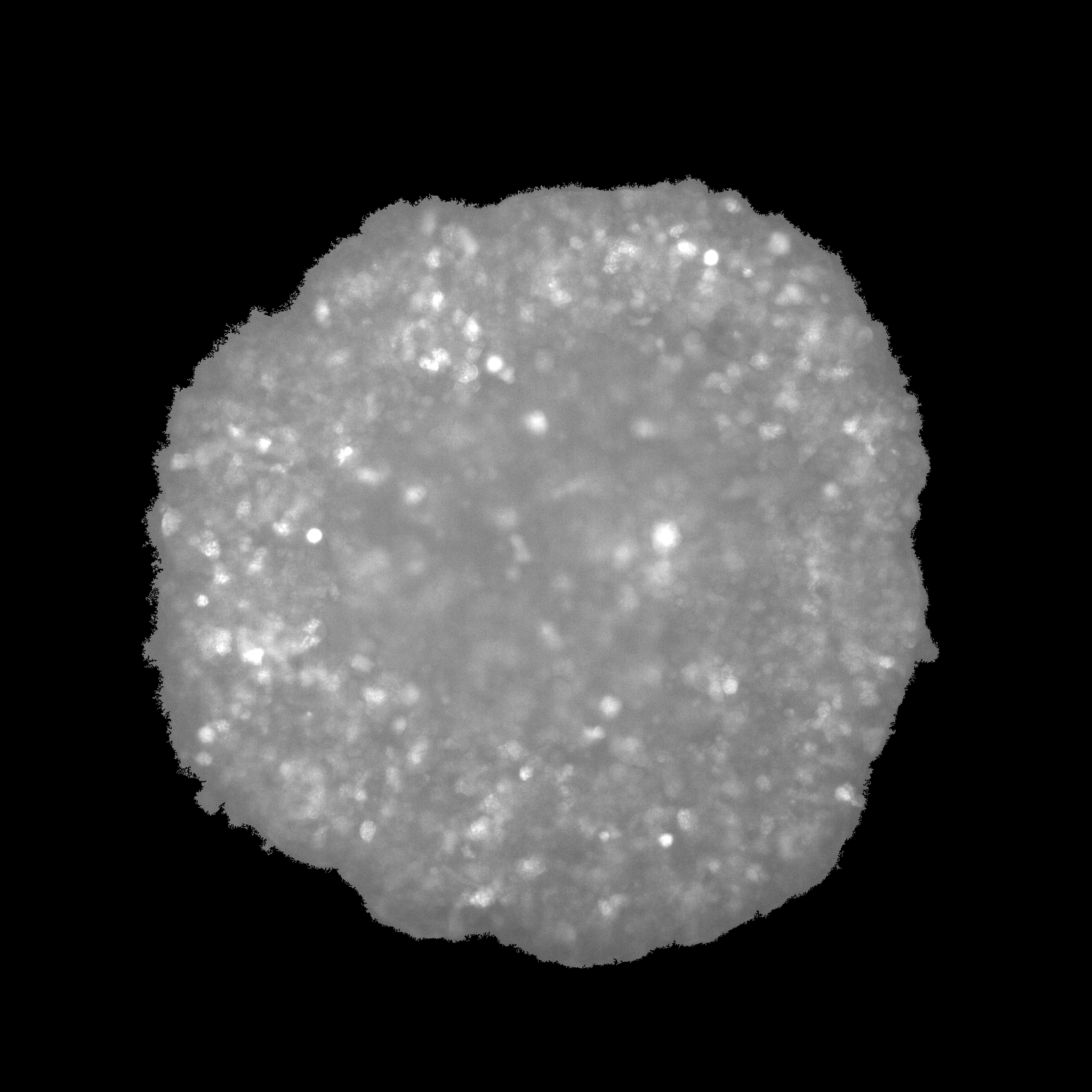

Supplement: Supplementary file 3 — Source Data Fig. 2 [file 44319_2023_52_MOESM3_ESM.zip › Figure 2/2A/405nm.tif]

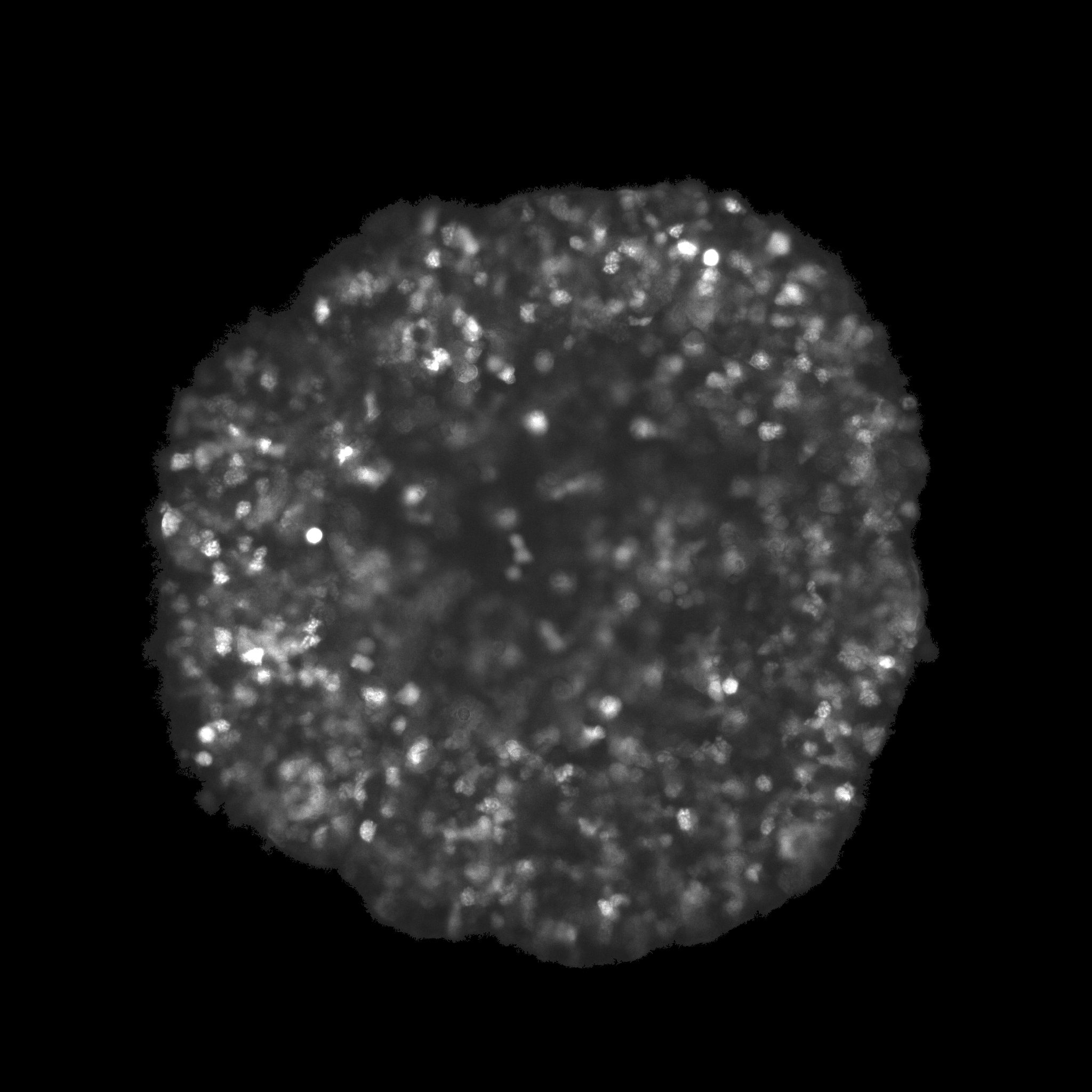

Supplement: Supplementary file 3 — Source Data Fig. 2 [file 44319_2023_52_MOESM3_ESM.zip › Figure 2/2A/488nm.tif]

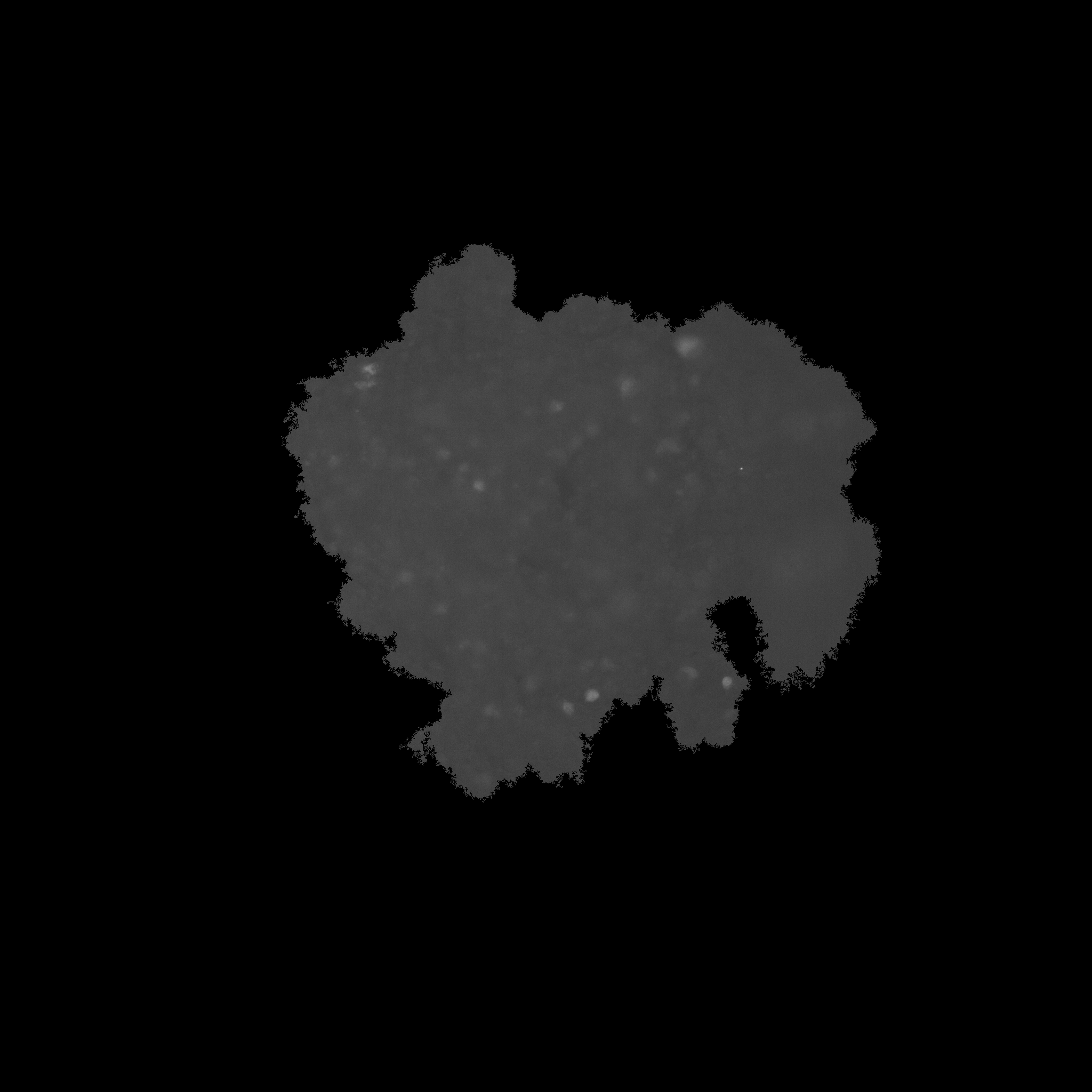

Supplement: Supplementary file 3 — Source Data Fig. 2 [file 44319_2023_52_MOESM3_ESM.zip › Figure 2/2D/B16-F10-405nm.tif]

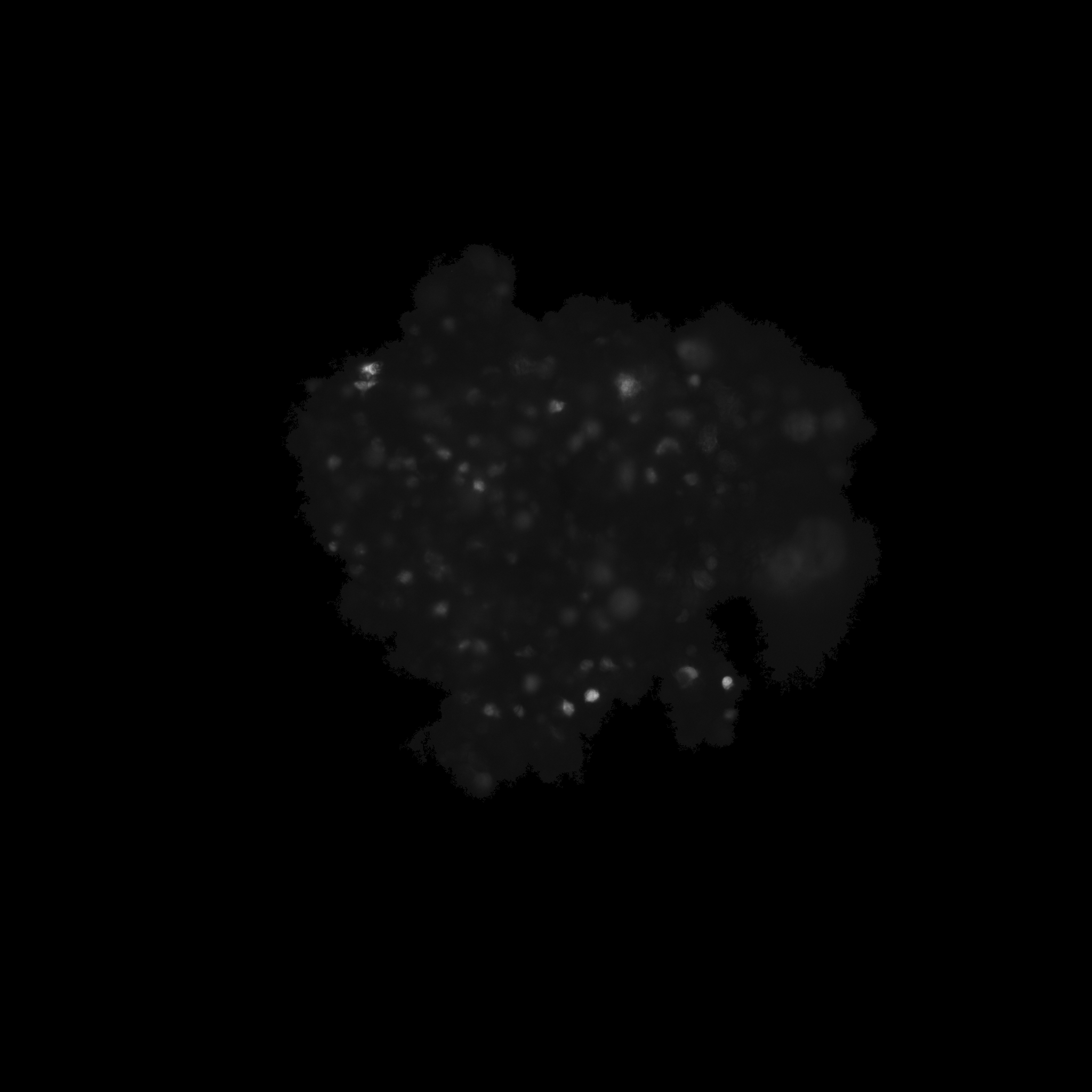

Supplement: Supplementary file 3 — Source Data Fig. 2 [file 44319_2023_52_MOESM3_ESM.zip › Figure 2/2D/B16-F10-488nm.tif]

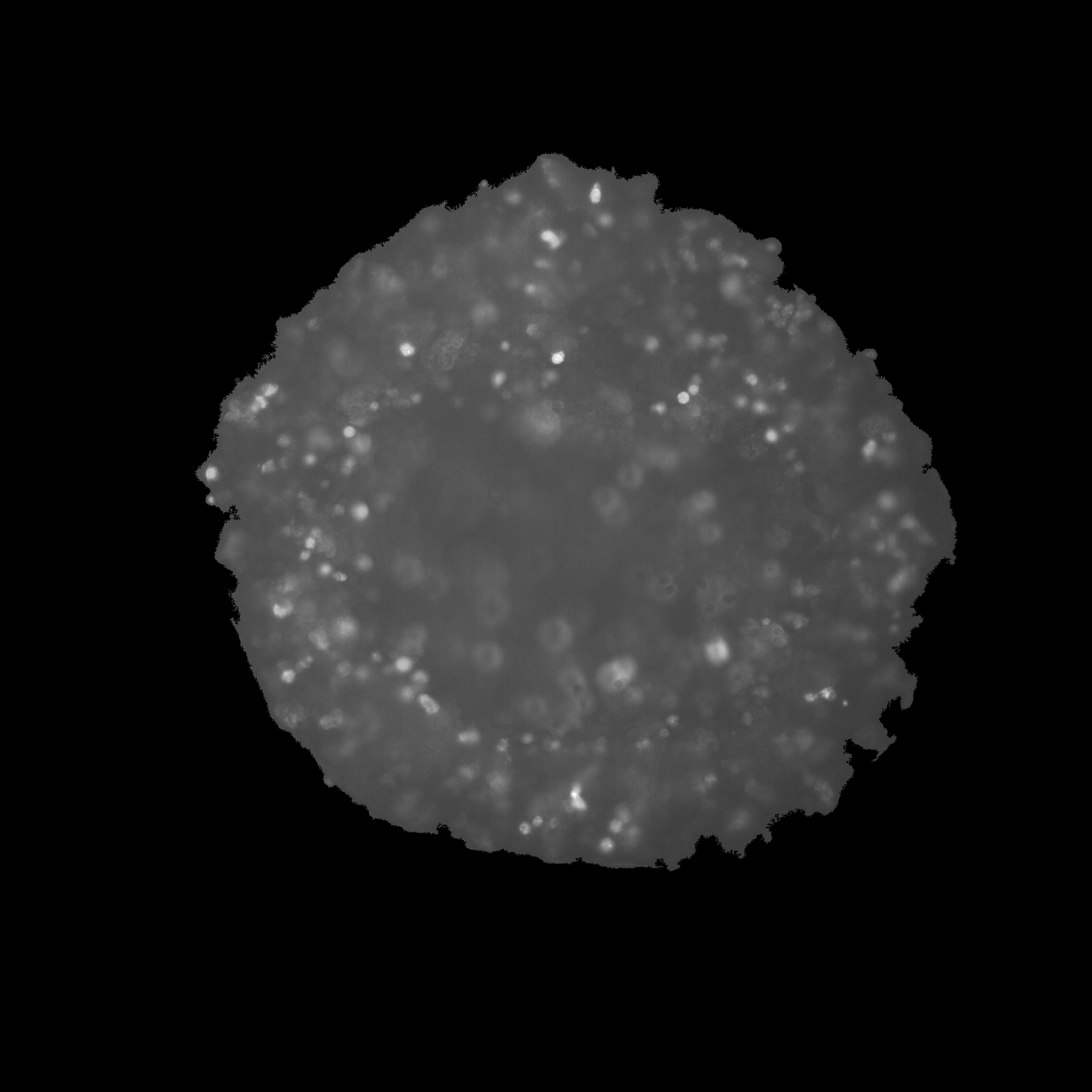

Supplement: Supplementary file 3 — Source Data Fig. 2 [file 44319_2023_52_MOESM3_ESM.zip › Figure 2/2D/HeLa-405nm.tif]

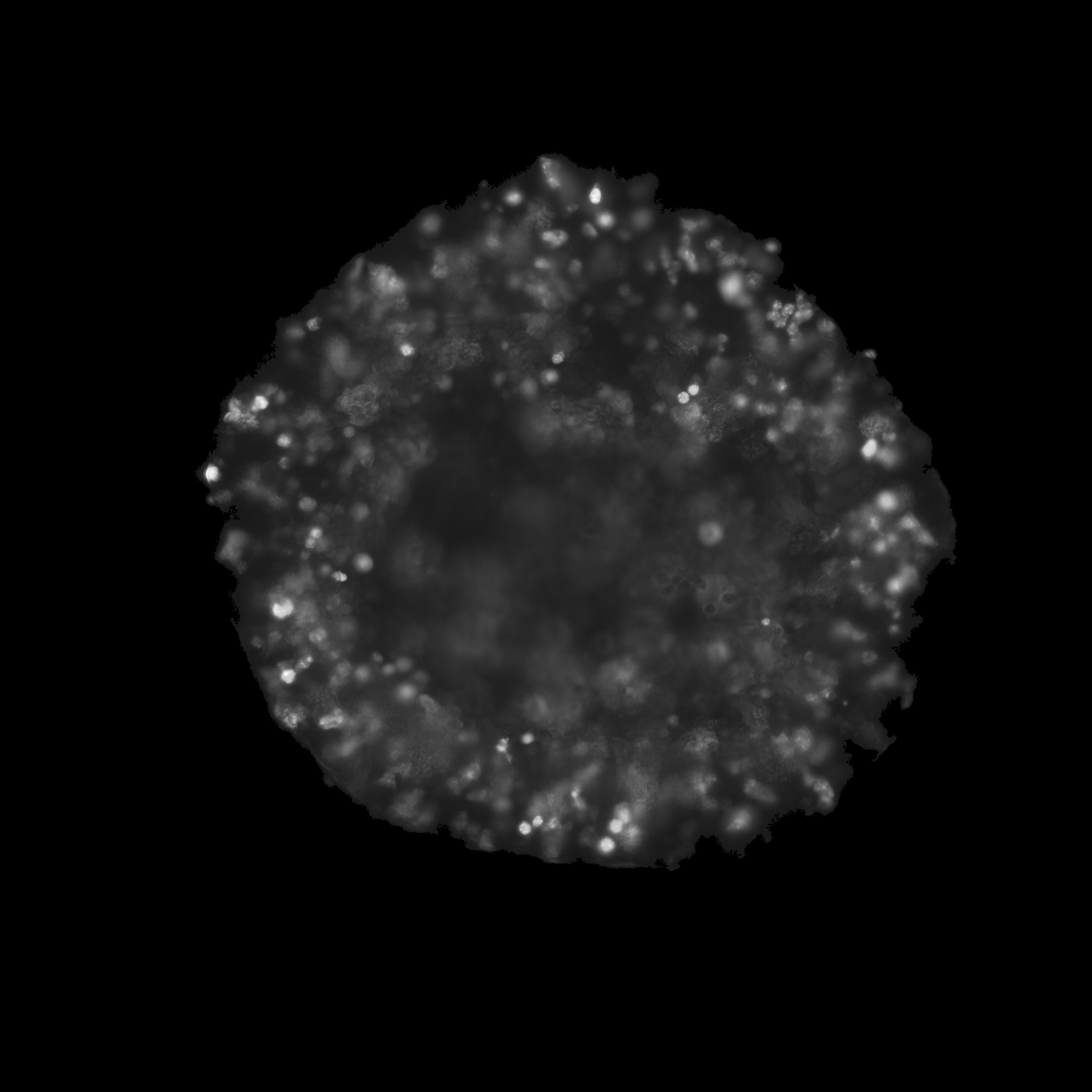

Supplement: Supplementary file 3 — Source Data Fig. 2 [file 44319_2023_52_MOESM3_ESM.zip › Figure 2/2D/HeLa-488nm.tif]

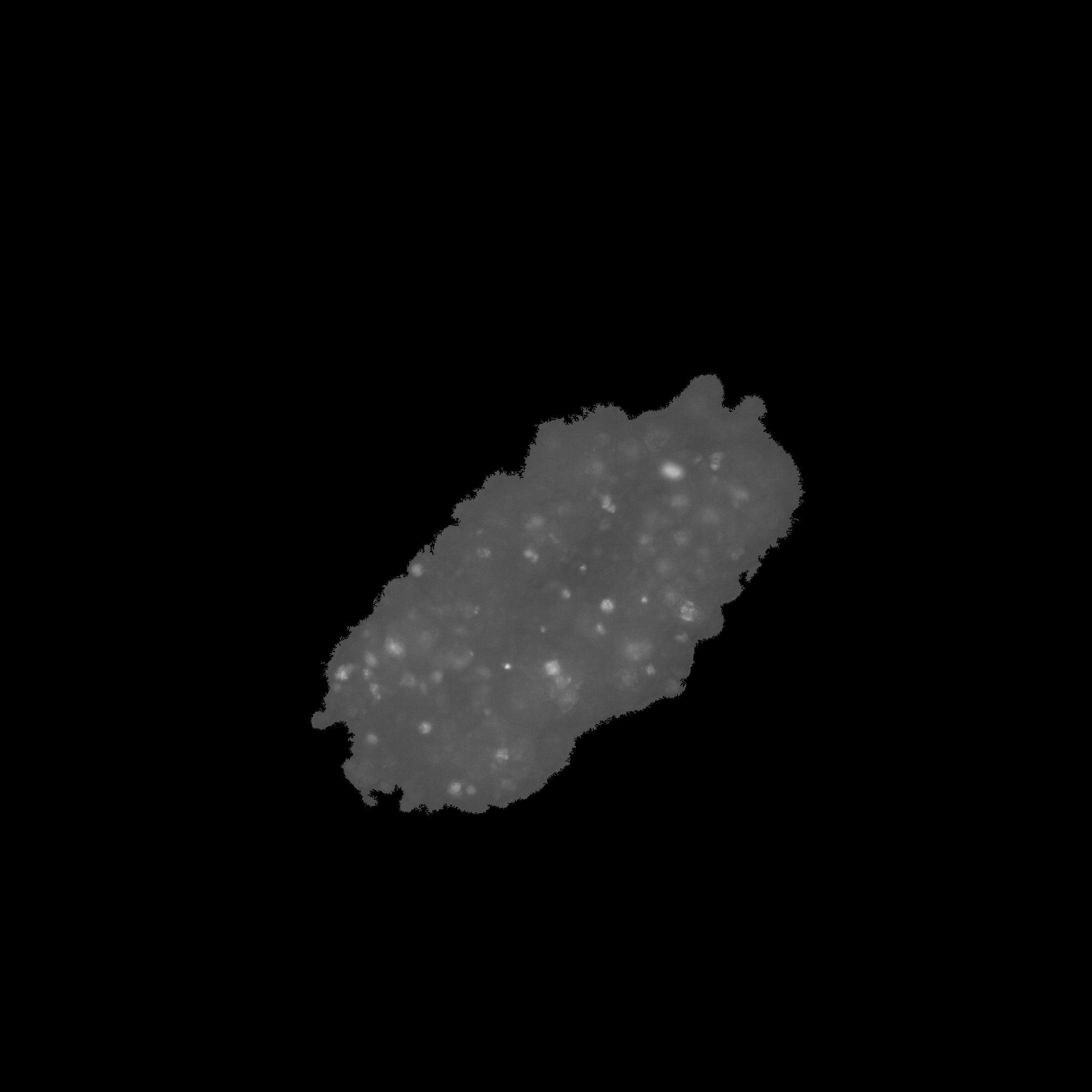

Supplement: Supplementary file 3 — Source Data Fig. 2 [file 44319_2023_52_MOESM3_ESM.zip › Figure 2/2D/MCF7-405nm.tif]

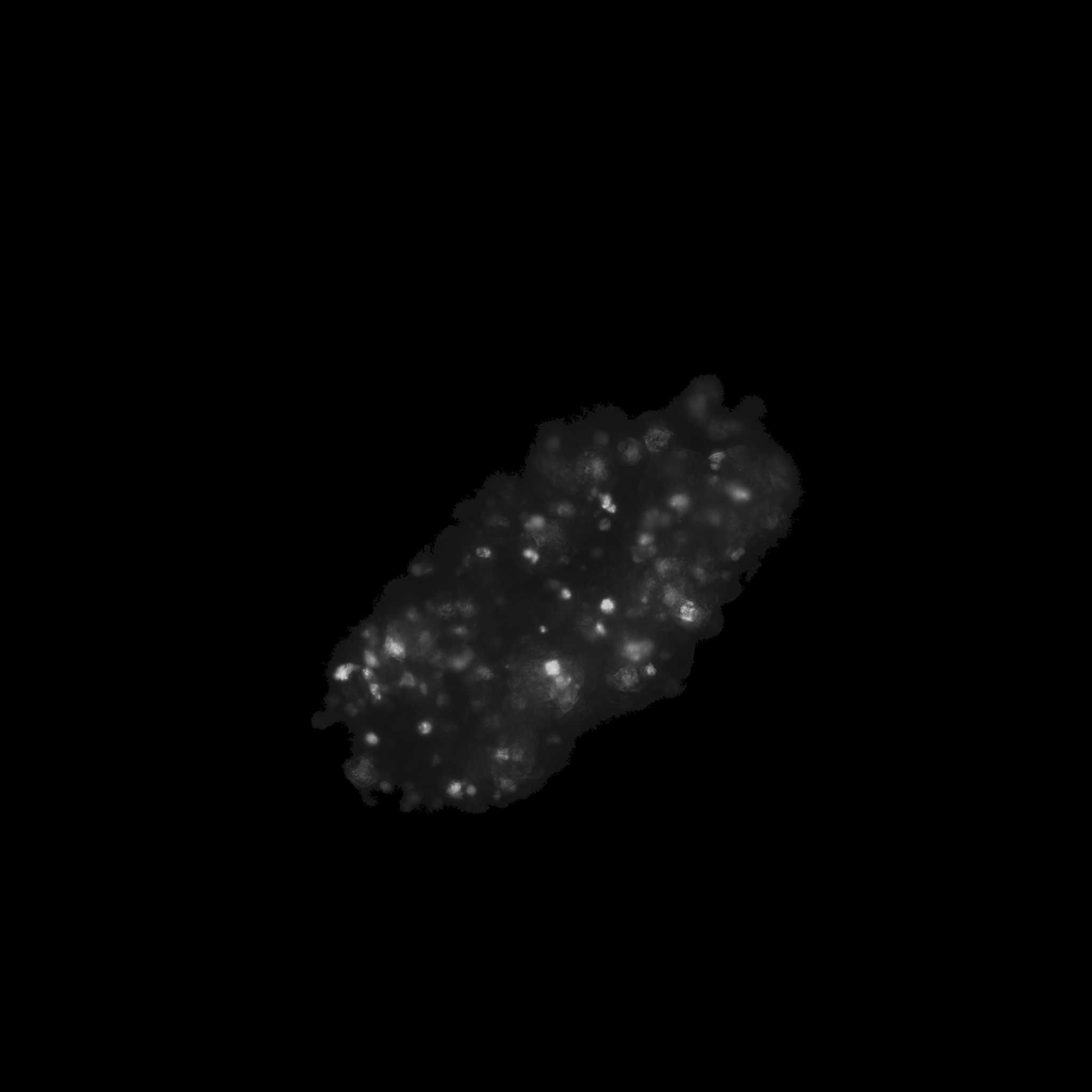

Supplement: Supplementary file 3 — Source Data Fig. 2 [file 44319_2023_52_MOESM3_ESM.zip › Figure 2/2D/MCF7-488nm.tif]

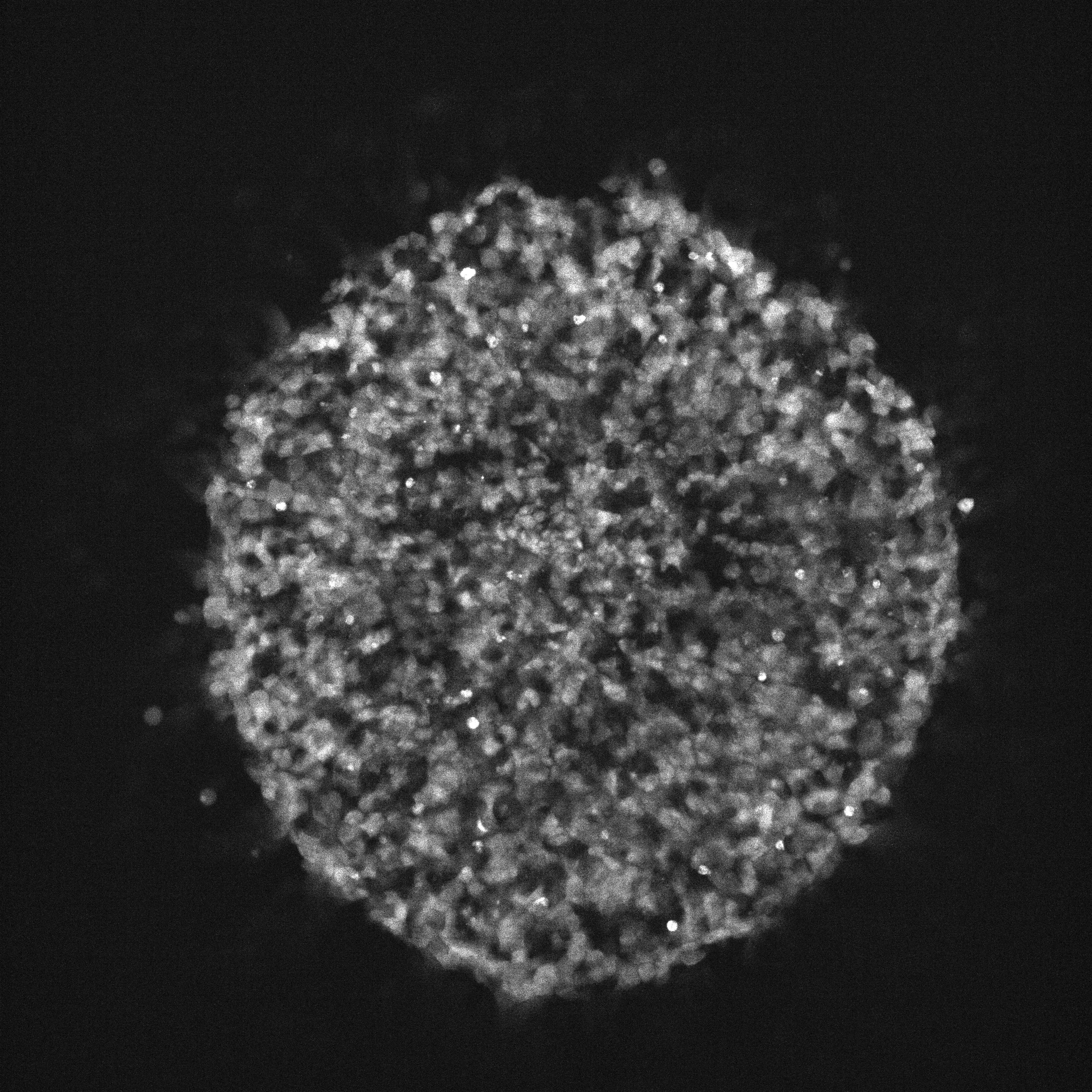

Supplement: Supplementary file 4 — Source Data Fig. 3 [file 44319_2023_52_MOESM4_ESM.zip › Figure 3/3D/mix spheroid in 2D.tif]

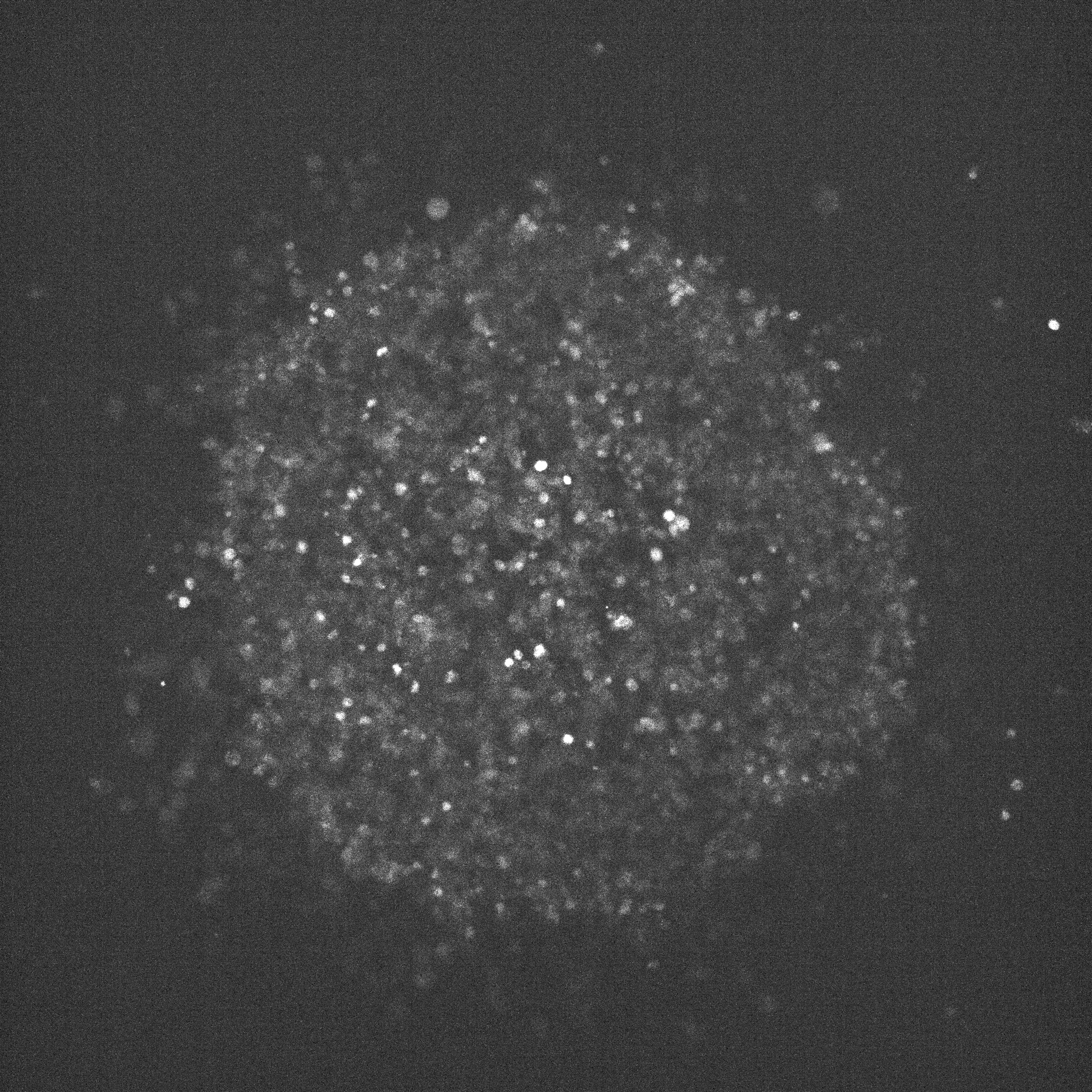

Supplement: Supplementary file 4 — Source Data Fig. 3 [file 44319_2023_52_MOESM4_ESM.zip › Figure 3/3F/mix spheroid in 3D.tif]

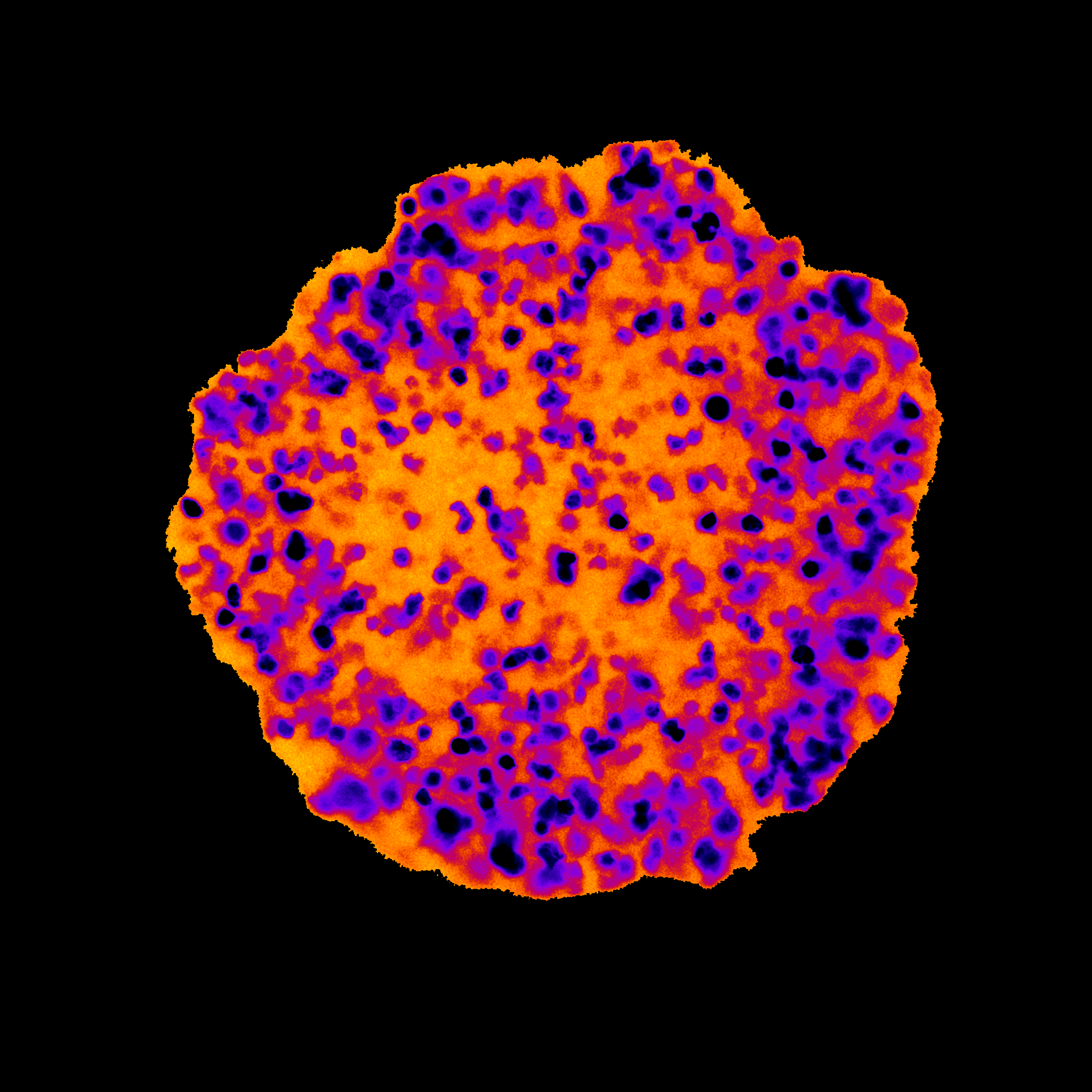

Supplement: Supplementary file 4 — Source Data Fig. 3 [file 44319_2023_52_MOESM4_ESM.zip › Figure 3/3I/RoGFP ratio WT+KO.tif]

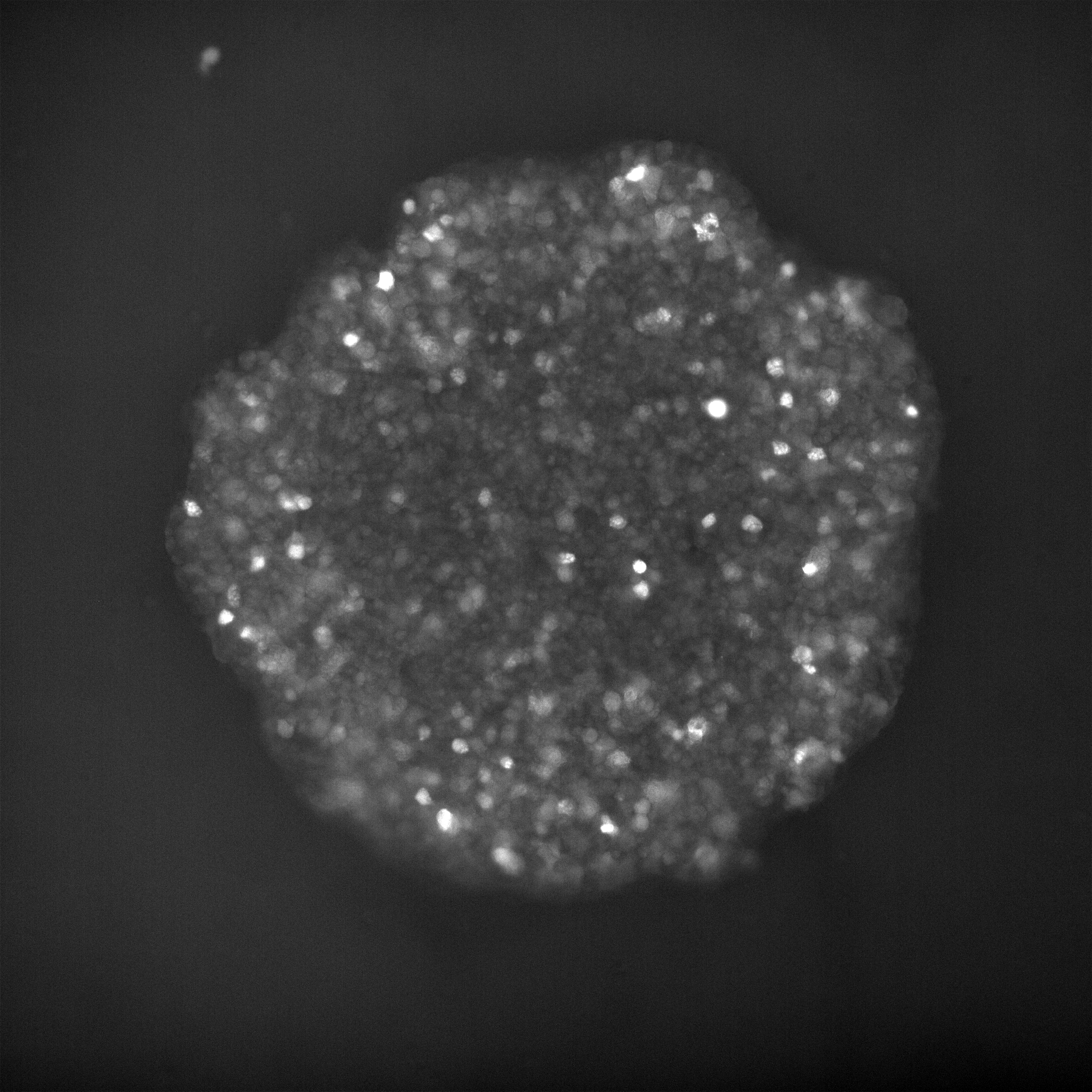

Supplement: Supplementary file 4 — Source Data Fig. 3 [file 44319_2023_52_MOESM4_ESM.zip › Figure 3/3I/WT+KO.tif]

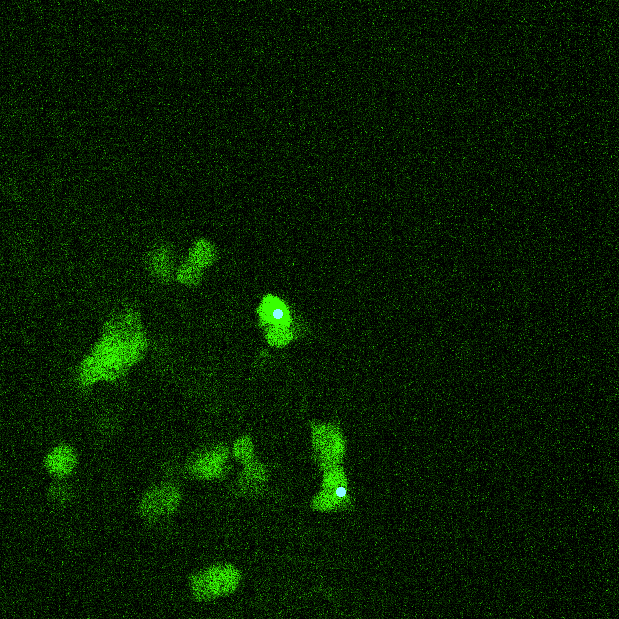

Supplement: Supplementary file 4 — Source Data Fig. 3 [file 44319_2023_52_MOESM4_ESM.zip › Figure 3/3L/WT+KO.tif]

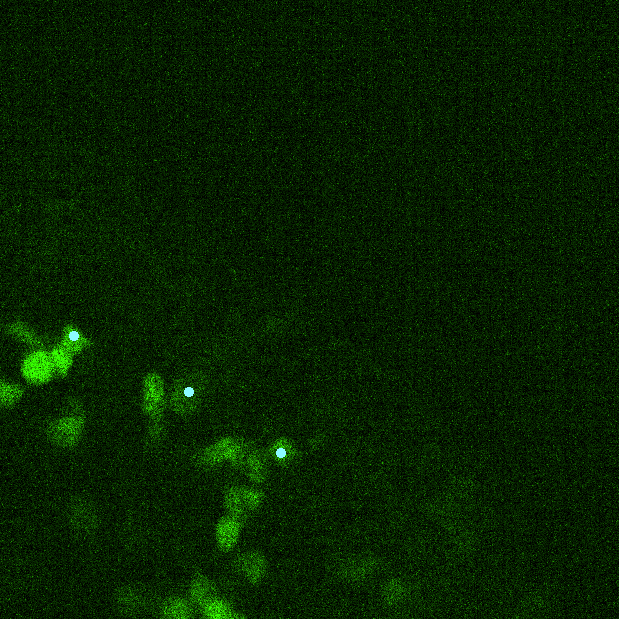

Supplement: Supplementary file 4 — Source Data Fig. 3 [file 44319_2023_52_MOESM4_ESM.zip › Figure 3/3L/WT+WT.tif]

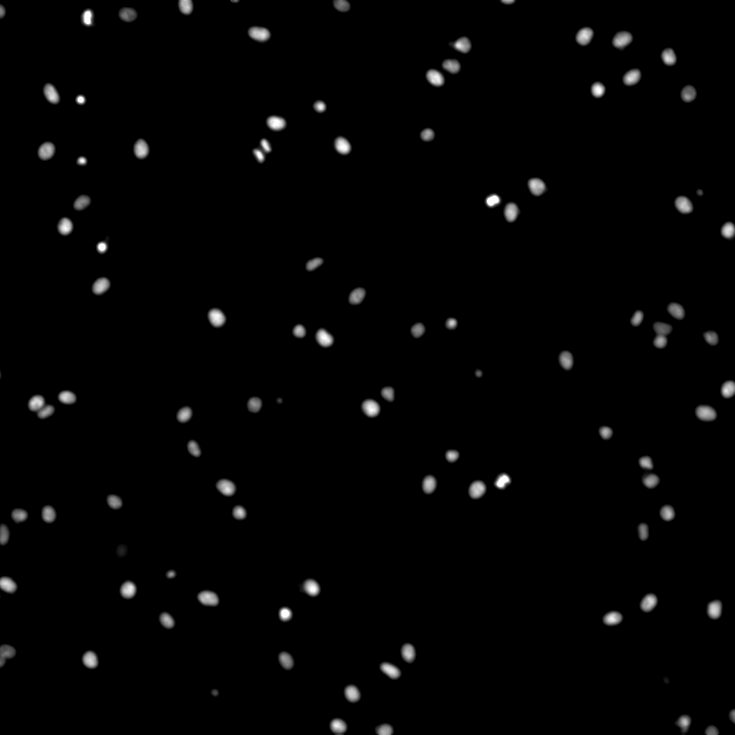

Supplement: Supplementary file 5 — Source Data Fig. 4 [file 44319_2023_52_MOESM5_ESM.zip › Figure 4/4C/ctrl.tif]

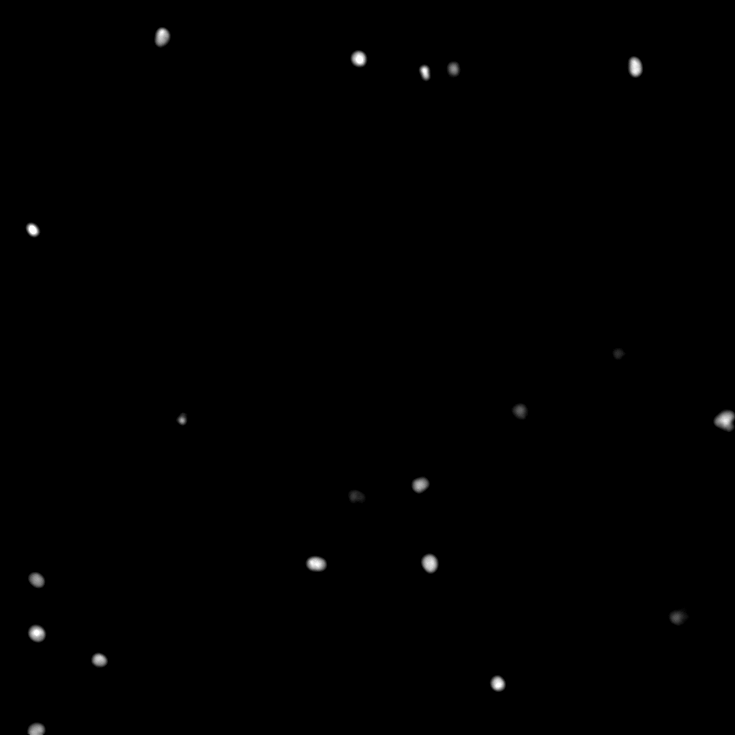

Supplement: Supplementary file 5 — Source Data Fig. 4 [file 44319_2023_52_MOESM5_ESM.zip › Figure 4/4C/H2O2+catalase.tif]

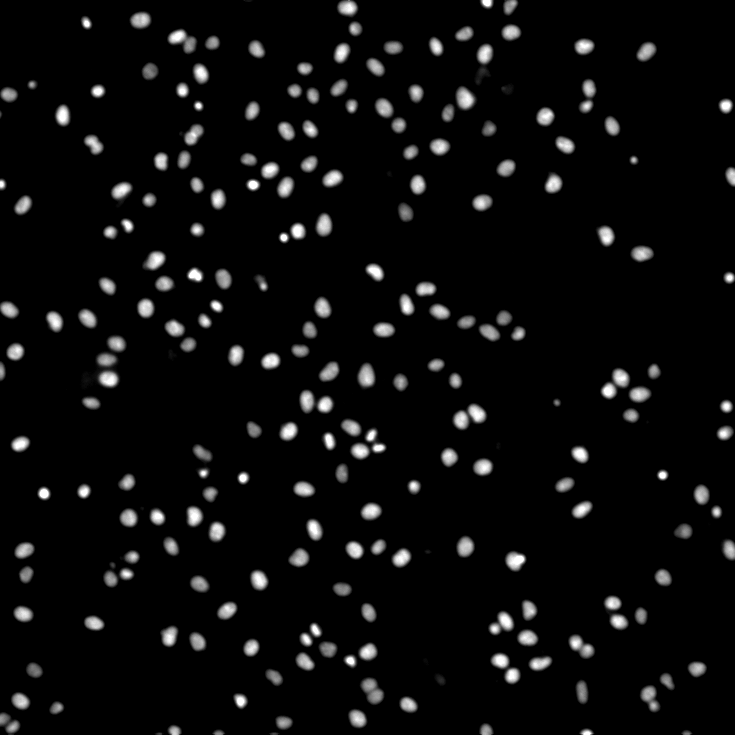

Supplement: Supplementary file 5 — Source Data Fig. 4 [file 44319_2023_52_MOESM5_ESM.zip › Figure 4/4C/H2O2.tif]

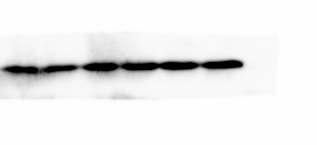

Supplement: Supplementary file 5 — Source Data Fig. 4 [file 44319_2023_52_MOESM5_ESM.zip › Figure 4/4H/GAPDH.tif]

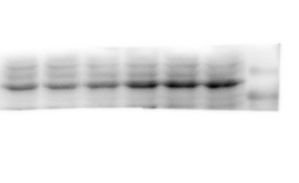

Supplement: Supplementary file 5 — Source Data Fig. 4 [file 44319_2023_52_MOESM5_ESM.zip › Figure 4/4H/pSrc.tif]

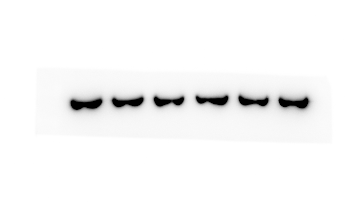

Supplement: Supplementary file 5 — Source Data Fig. 4 [file 44319_2023_52_MOESM5_ESM.zip › Figure 4/4H/Total src.tif]

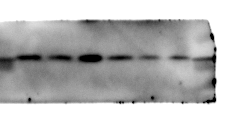

Supplement: Supplementary file 5 — Source Data Fig. 4 [file 44319_2023_52_MOESM5_ESM.zip › Figure 4/4J/active RhoA.tif]

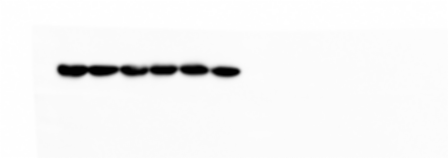

Supplement: Supplementary file 5 — Source Data Fig. 4 [file 44319_2023_52_MOESM5_ESM.zip › Figure 4/4J/GAPDH.tif]

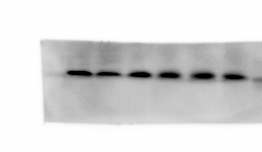

Supplement: Supplementary file 5 — Source Data Fig. 4 [file 44319_2023_52_MOESM5_ESM.zip › Figure 4/4J/Total RhoA.tif]

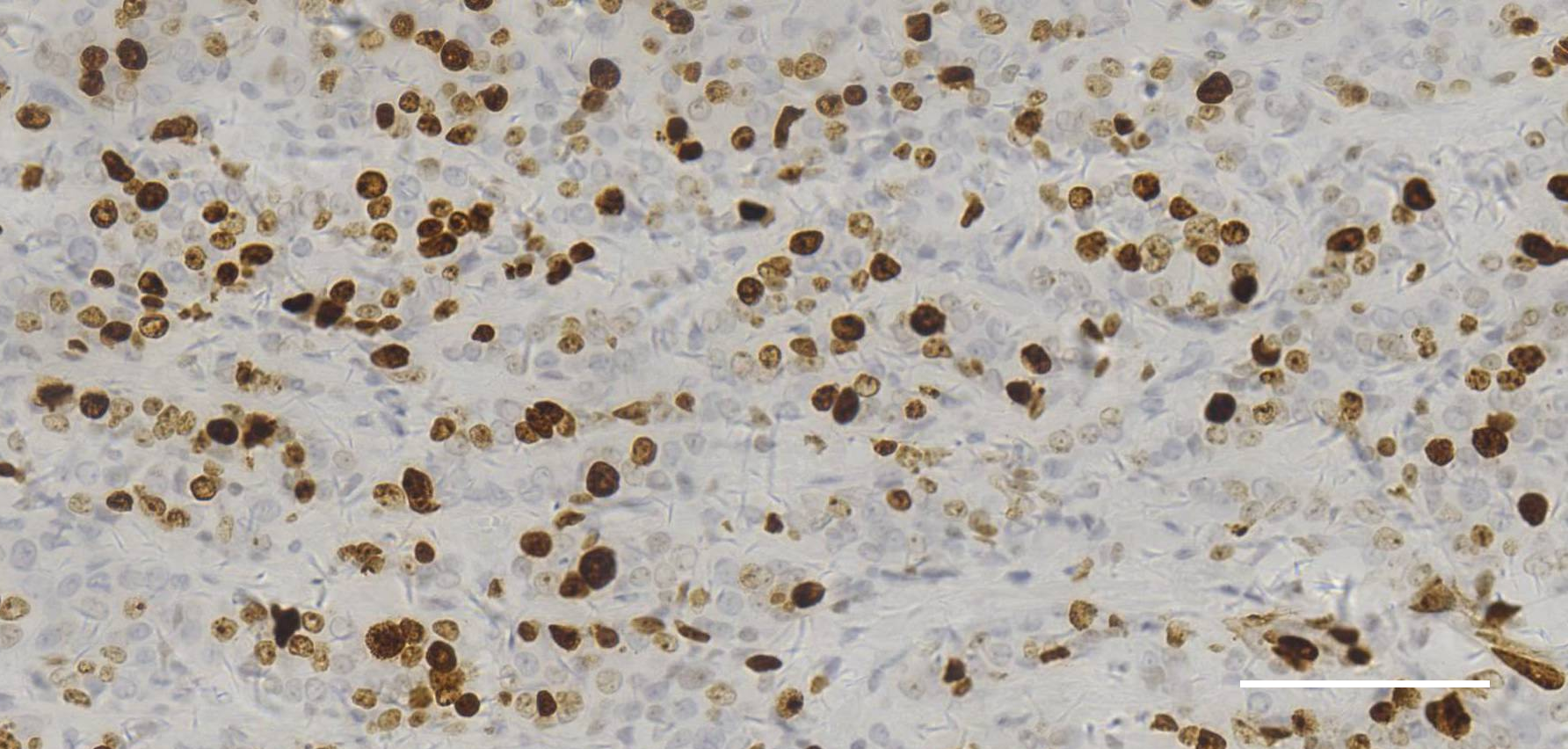

Supplement: Supplementary file 6 — Figure EV Source Data [file 44319_2023_52_MOESM6_ESM.zip › Figure EV/Figure EV1/1C/L group.tif]

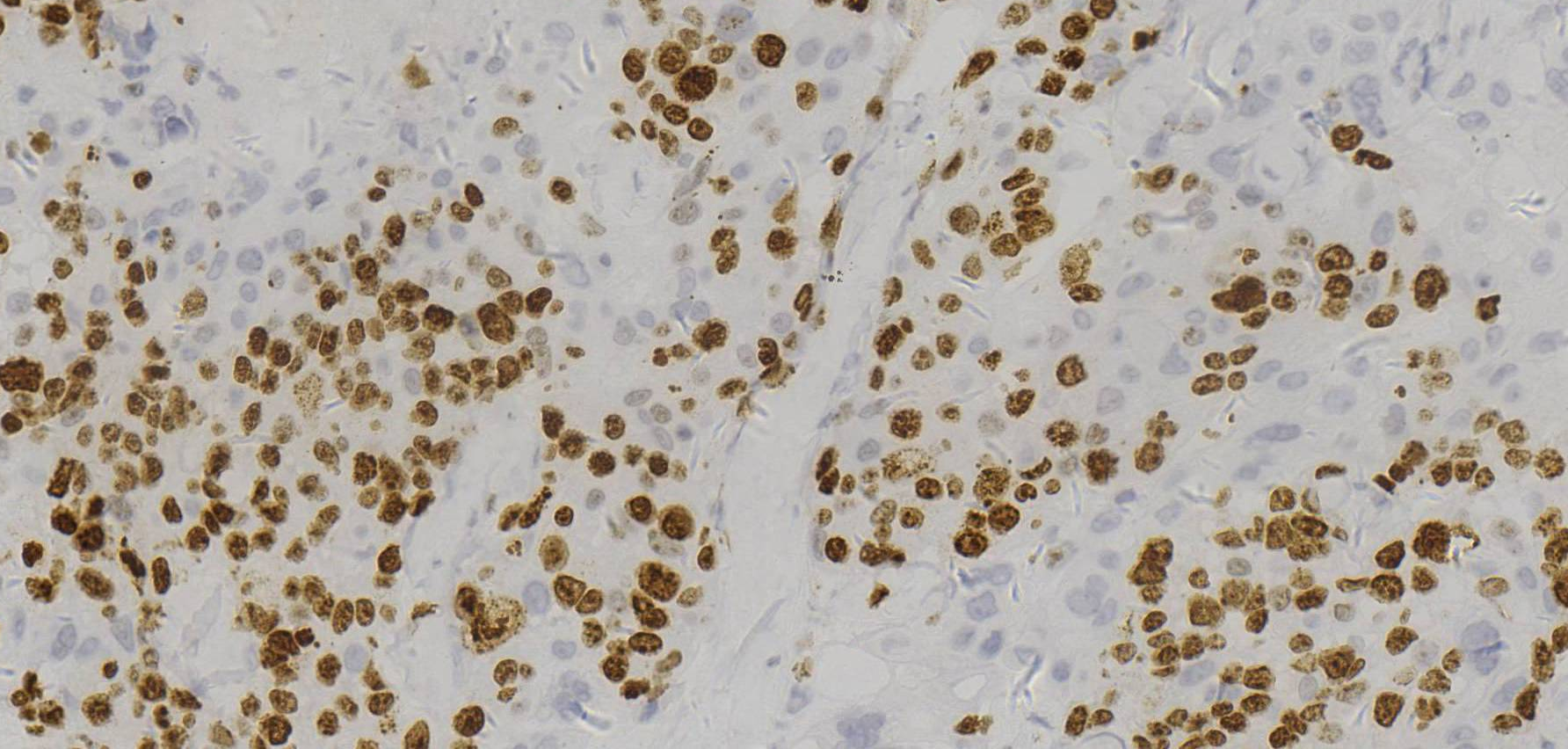

Supplement: Supplementary file 6 — Figure EV Source Data [file 44319_2023_52_MOESM6_ESM.zip › Figure EV/Figure EV1/1C/M group.tif]

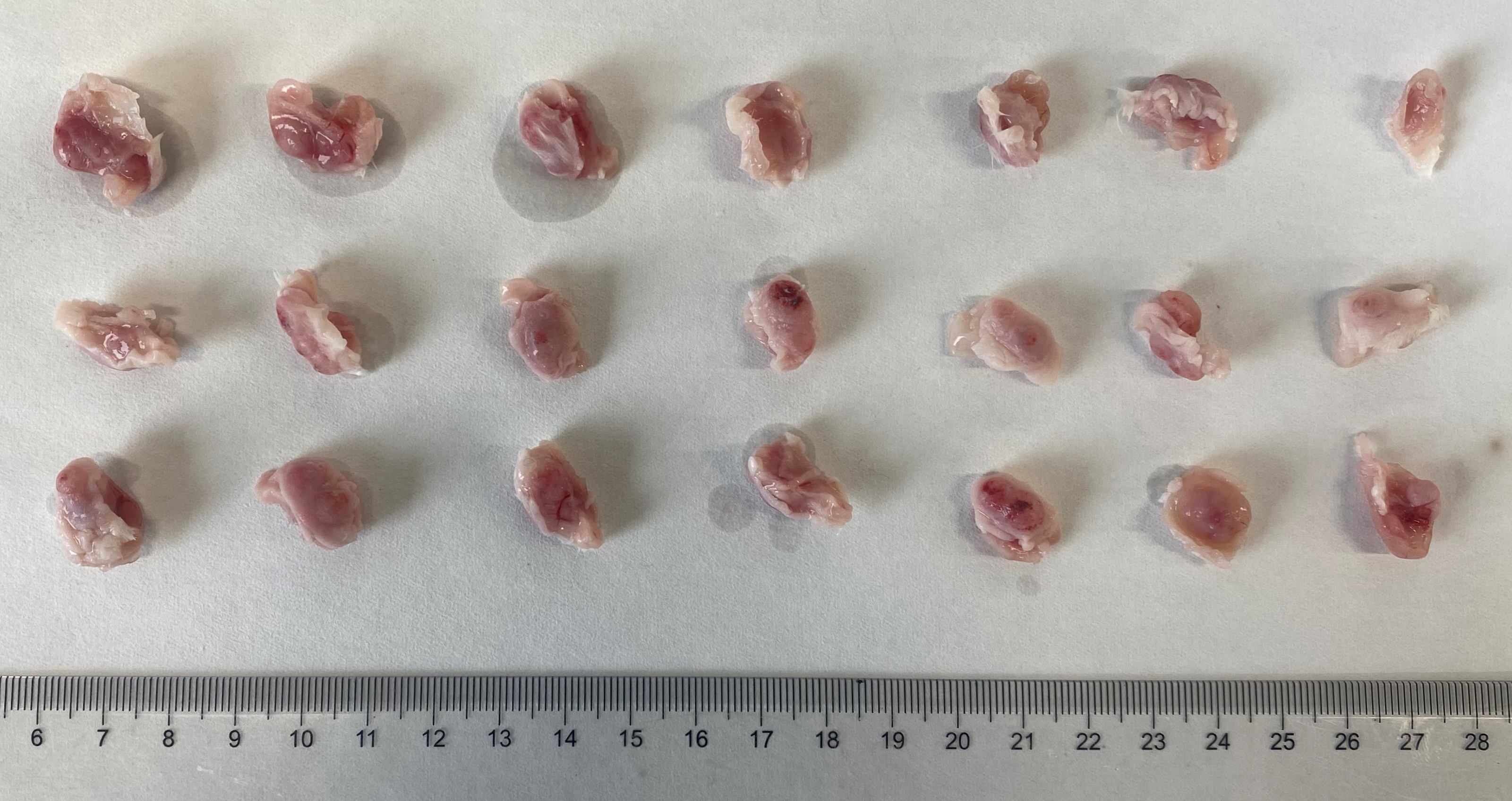

Supplement: Supplementary file 6 — Figure EV Source Data [file 44319_2023_52_MOESM6_ESM.zip › Figure EV/Figure EV1/1E/tumor.jpg]

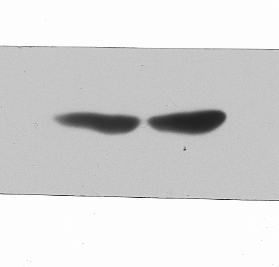

Supplement: Supplementary file 6 — Figure EV Source Data [file 44319_2023_52_MOESM6_ESM.zip › Figure EV/Figure EV2/2A/IMS-RoGFP-GAPDH.tif]

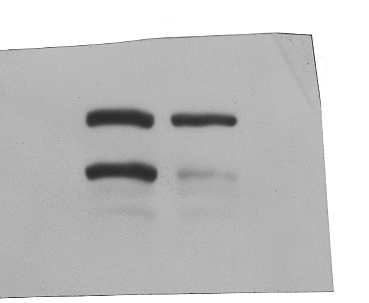

Supplement: Supplementary file 6 — Figure EV Source Data [file 44319_2023_52_MOESM6_ESM.zip › Figure EV/Figure EV2/2A/IMS-RoGFP-Myo19.tif]

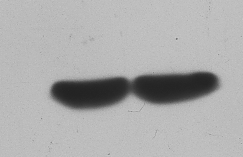

Supplement: Supplementary file 6 — Figure EV Source Data [file 44319_2023_52_MOESM6_ESM.zip › Figure EV/Figure EV2/2A/matirx-RoGFP-GAPDH.tif]

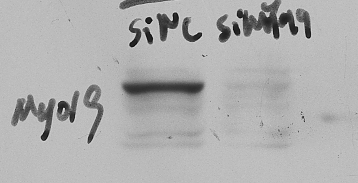

Supplement: Supplementary file 6 — Figure EV Source Data [file 44319_2023_52_MOESM6_ESM.zip › Figure EV/Figure EV2/2A/matrix-RoGFP-Myo19.tif]

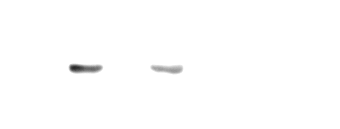

Supplement: Supplementary file 6 — Figure EV Source Data [file 44319_2023_52_MOESM6_ESM.zip › Figure EV/Figure EV2/2E/GAPDH.tif]

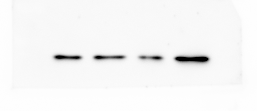

Supplement: Supplementary file 6 — Figure EV Source Data [file 44319_2023_52_MOESM6_ESM.zip › Figure EV/Figure EV2/2E/Tom20.tif]

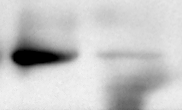

Supplement: Supplementary file 6 — Figure EV Source Data [file 44319_2023_52_MOESM6_ESM.zip › Figure EV/Figure EV2/2K/GAPDH.tif]

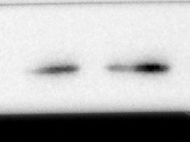

Supplement: Supplementary file 6 — Figure EV Source Data [file 44319_2023_52_MOESM6_ESM.zip › Figure EV/Figure EV2/2K/Nrf2.tif]

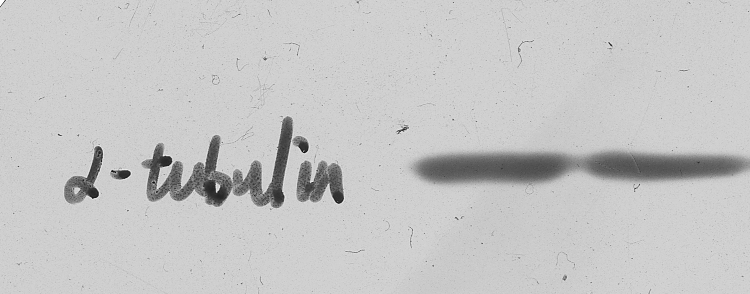

Supplement: Supplementary file 6 — Figure EV Source Data [file 44319_2023_52_MOESM6_ESM.zip › Figure EV/Figure EV2/2N/a tubulin.tif]

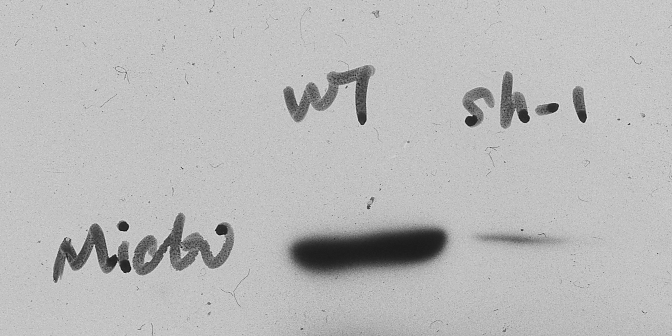

Supplement: Supplementary file 6 — Figure EV Source Data [file 44319_2023_52_MOESM6_ESM.zip › Figure EV/Figure EV2/2N/Mic60.tif]

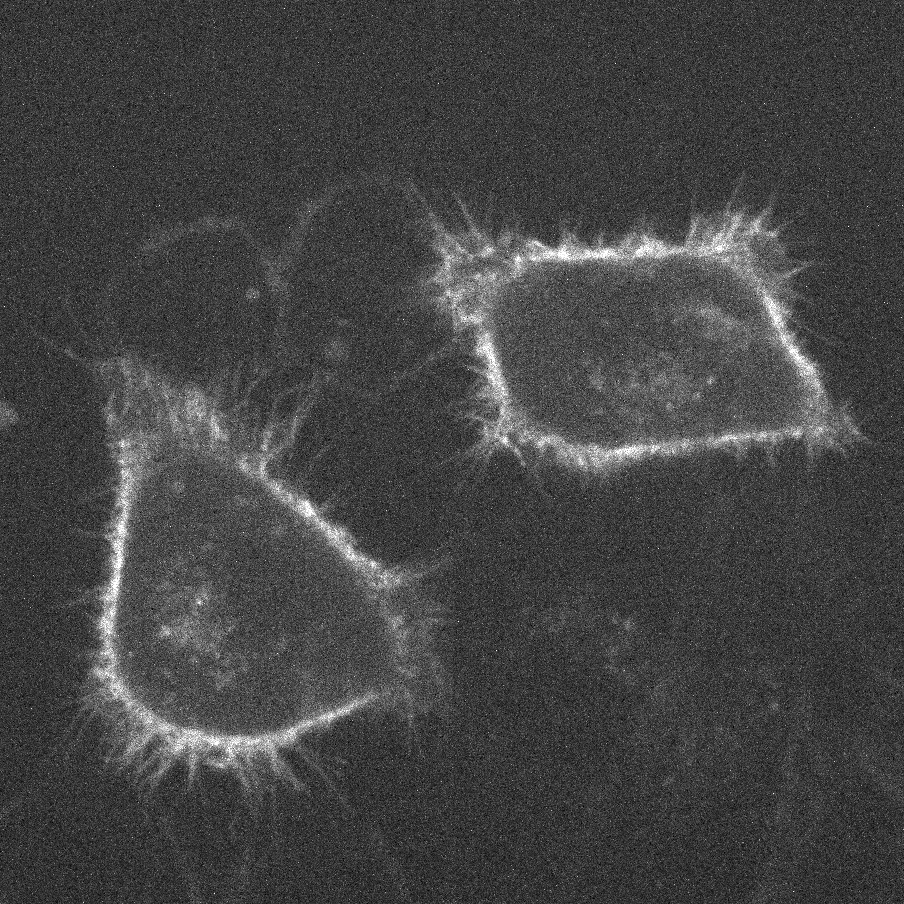

Supplement: Supplementary file 6 — Figure EV Source Data [file 44319_2023_52_MOESM6_ESM.zip › Figure EV/Figure EV3/2B/ss-HyPer-GPI.tif]

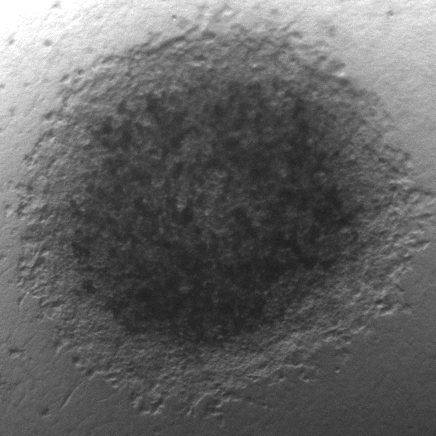

Supplement: Supplementary file 6 — Figure EV Source Data [file 44319_2023_52_MOESM6_ESM.zip › Figure EV/Figure EV4/4A/3D invasion.tif]

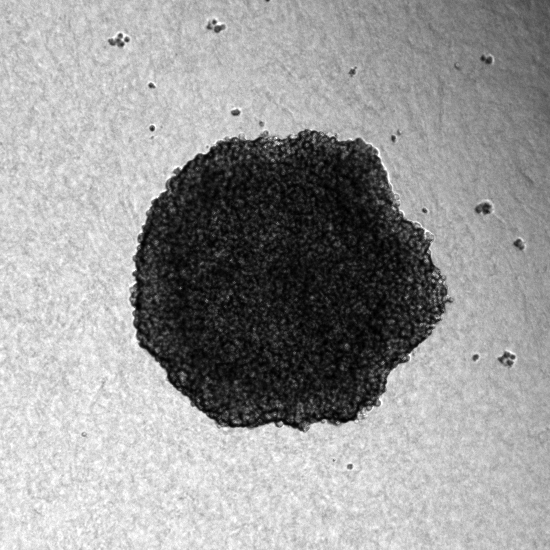

Supplement: Supplementary file 6 — Figure EV Source Data [file 44319_2023_52_MOESM6_ESM.zip › Figure EV/Figure EV4/4B/Myo19 KO+catalase-0h.tif]

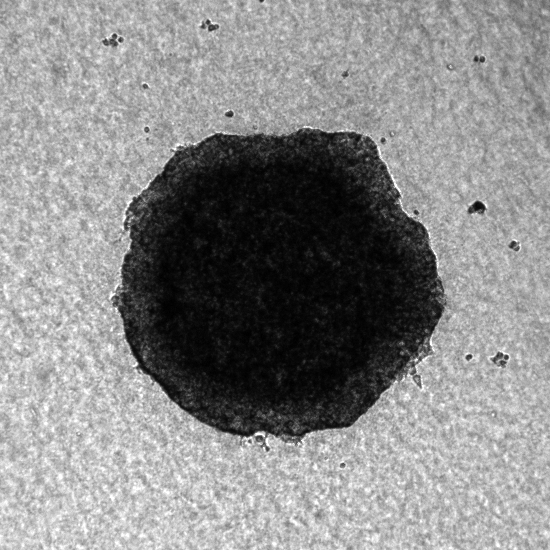

Supplement: Supplementary file 6 — Figure EV Source Data [file 44319_2023_52_MOESM6_ESM.zip › Figure EV/Figure EV4/4B/Myo19 KO+catalase-48h.tif]

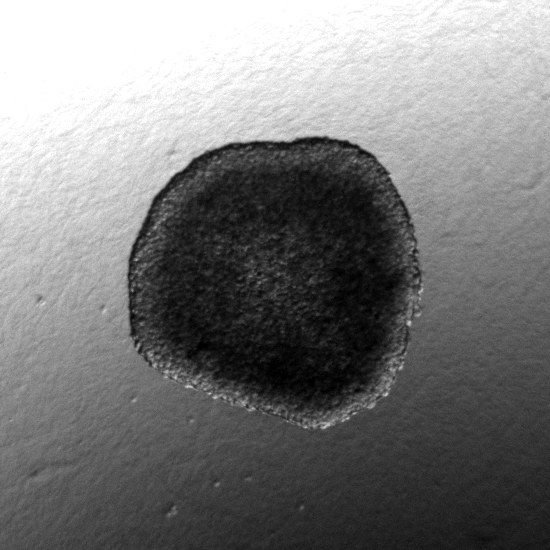

Supplement: Supplementary file 6 — Figure EV Source Data [file 44319_2023_52_MOESM6_ESM.zip › Figure EV/Figure EV4/4B/Myo19 KO-0h.tif]

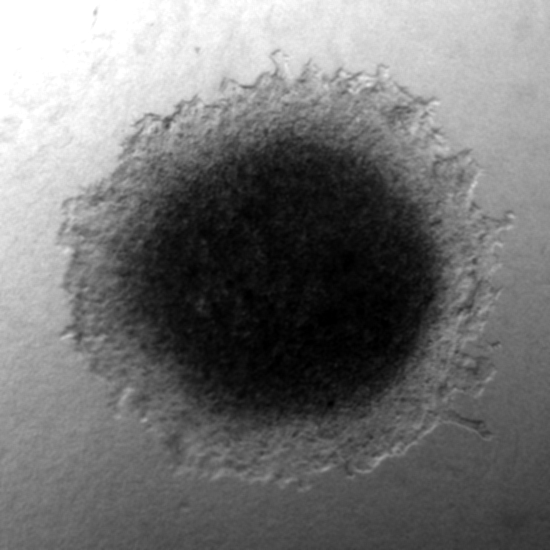

Supplement: Supplementary file 6 — Figure EV Source Data [file 44319_2023_52_MOESM6_ESM.zip › Figure EV/Figure EV4/4B/Myo19 KO-48h.tif]

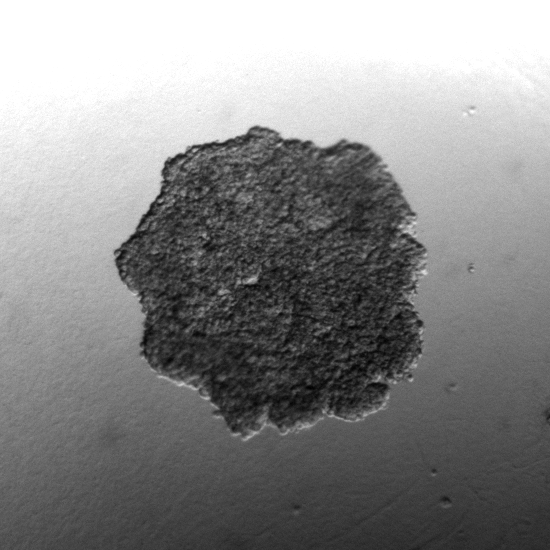

Supplement: Supplementary file 6 — Figure EV Source Data [file 44319_2023_52_MOESM6_ESM.zip › Figure EV/Figure EV4/4B/Nrf2 KD-0h.tif]

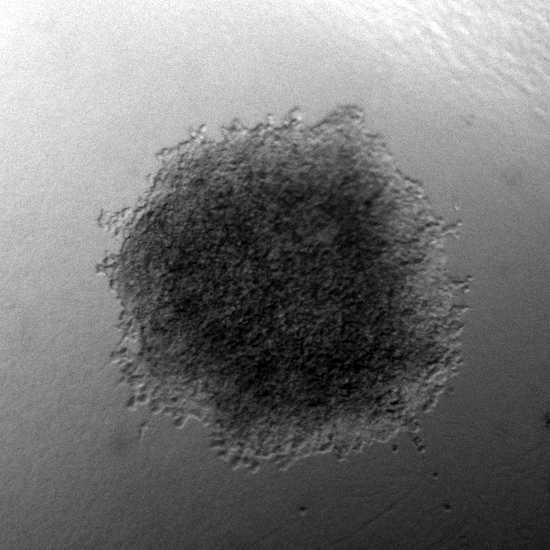

Supplement: Supplementary file 6 — Figure EV Source Data [file 44319_2023_52_MOESM6_ESM.zip › Figure EV/Figure EV4/4B/Nrf2 KD-48h.tif]

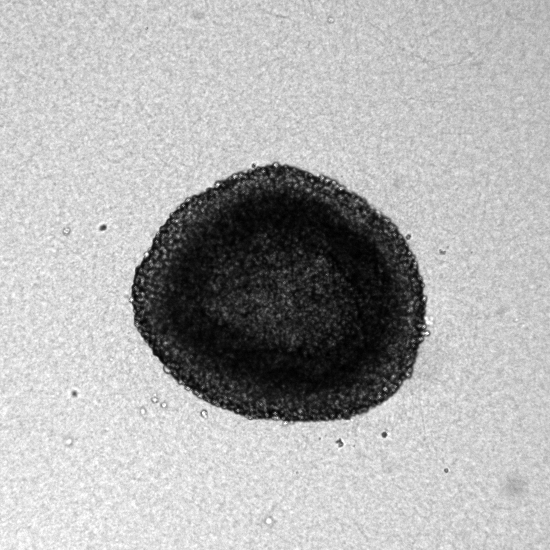

Supplement: Supplementary file 6 — Figure EV Source Data [file 44319_2023_52_MOESM6_ESM.zip › Figure EV/Figure EV4/4B/WT+catalase-0h.tif]

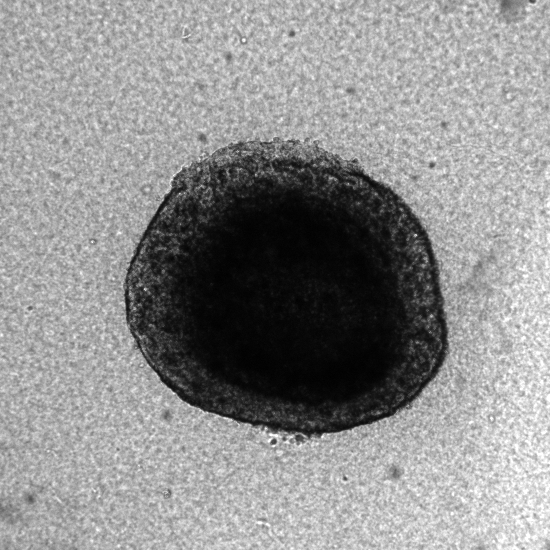

Supplement: Supplementary file 6 — Figure EV Source Data [file 44319_2023_52_MOESM6_ESM.zip › Figure EV/Figure EV4/4B/WT+catalase-48h.tif]

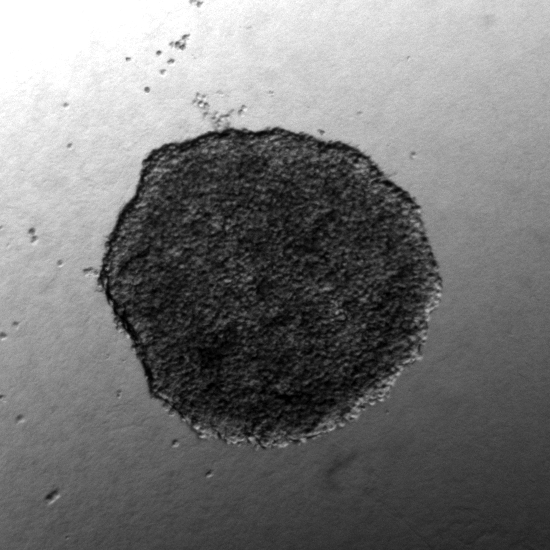

Supplement: Supplementary file 6 — Figure EV Source Data [file 44319_2023_52_MOESM6_ESM.zip › Figure EV/Figure EV4/4B/WT-0h.tif]

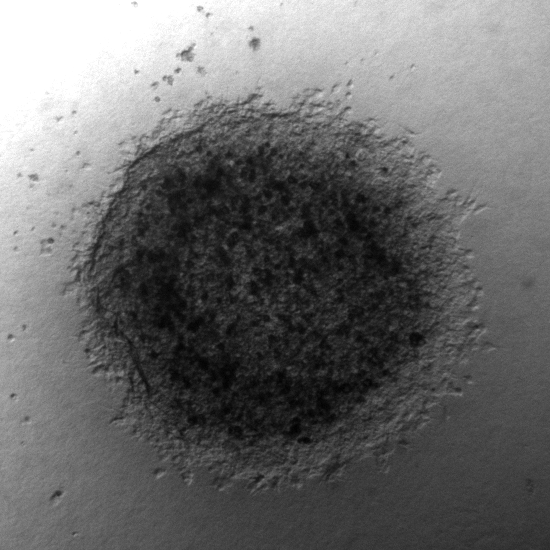

Supplement: Supplementary file 6 — Figure EV Source Data [file 44319_2023_52_MOESM6_ESM.zip › Figure EV/Figure EV4/4B/WT-48h.tif]

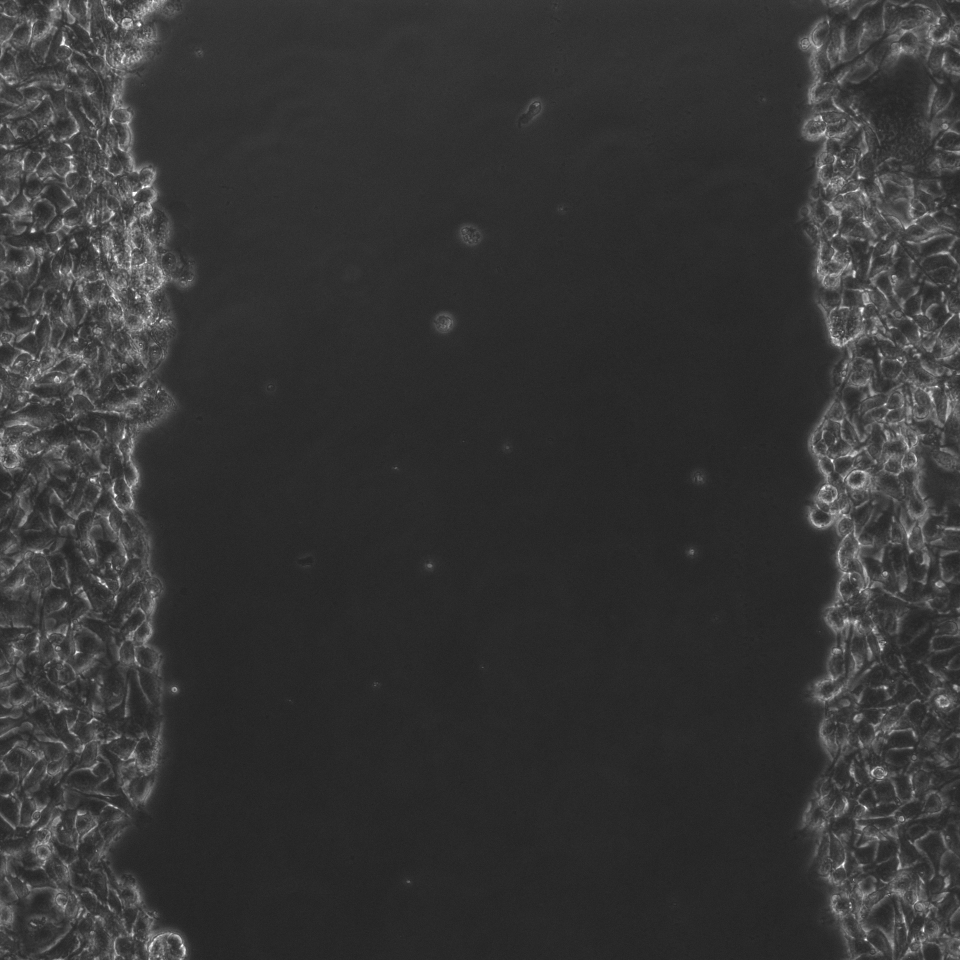

Supplement: Supplementary file 6 — Figure EV Source Data [file 44319_2023_52_MOESM6_ESM.zip › Figure EV/Figure EV4/4F/Myo19 KD-0h.tif]

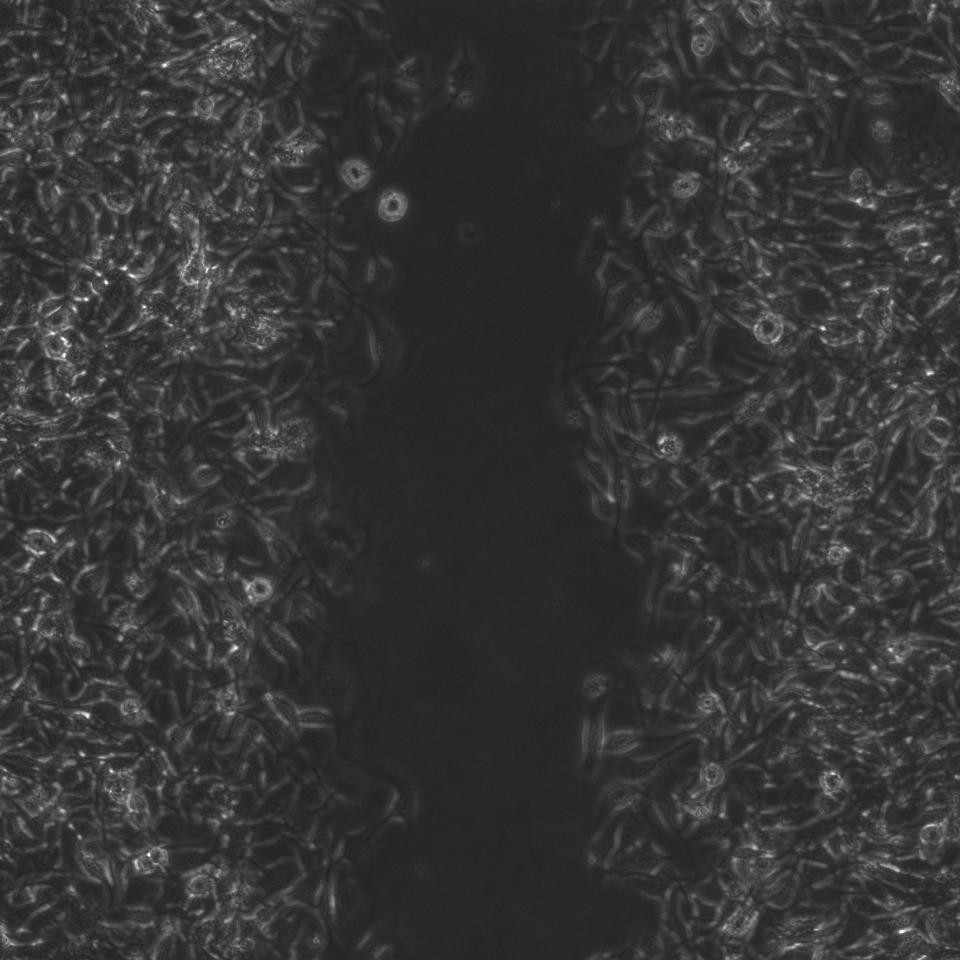

Supplement: Supplementary file 6 — Figure EV Source Data [file 44319_2023_52_MOESM6_ESM.zip › Figure EV/Figure EV4/4F/Myo19 KD-16h.tif]

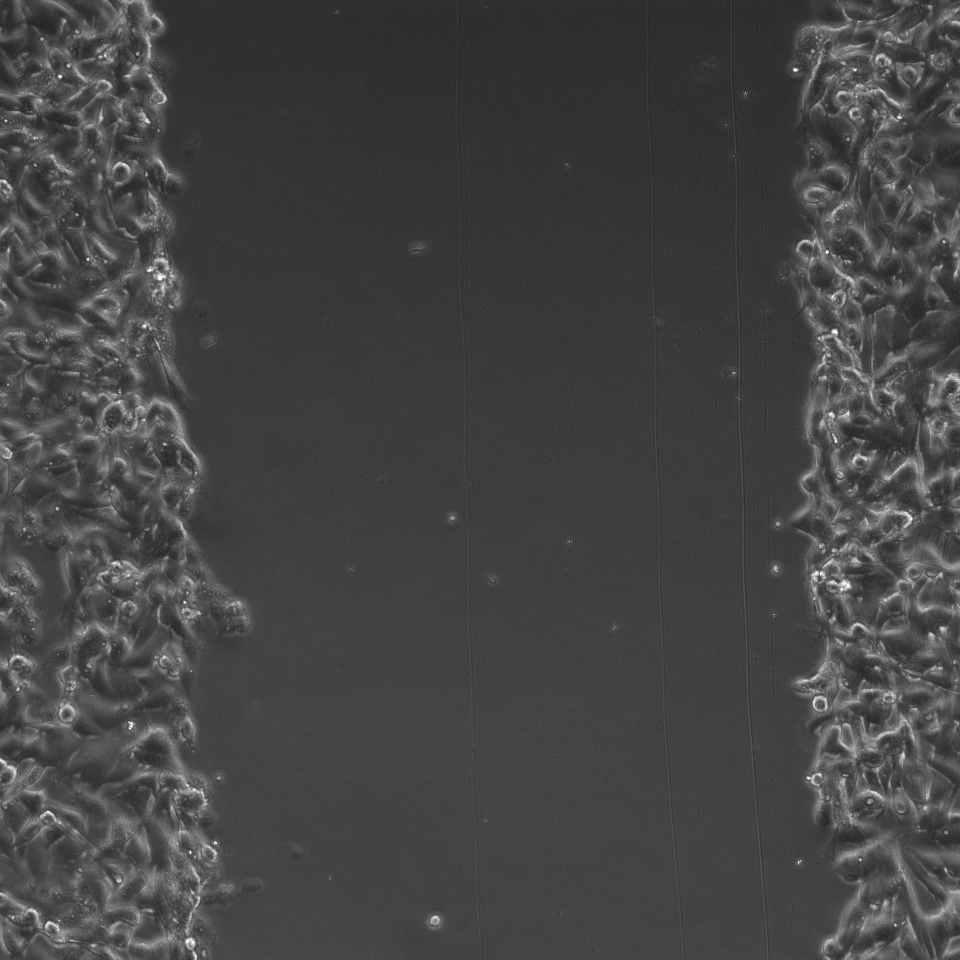

Supplement: Supplementary file 6 — Figure EV Source Data [file 44319_2023_52_MOESM6_ESM.zip › Figure EV/Figure EV4/4F/scramble-0h.tif]

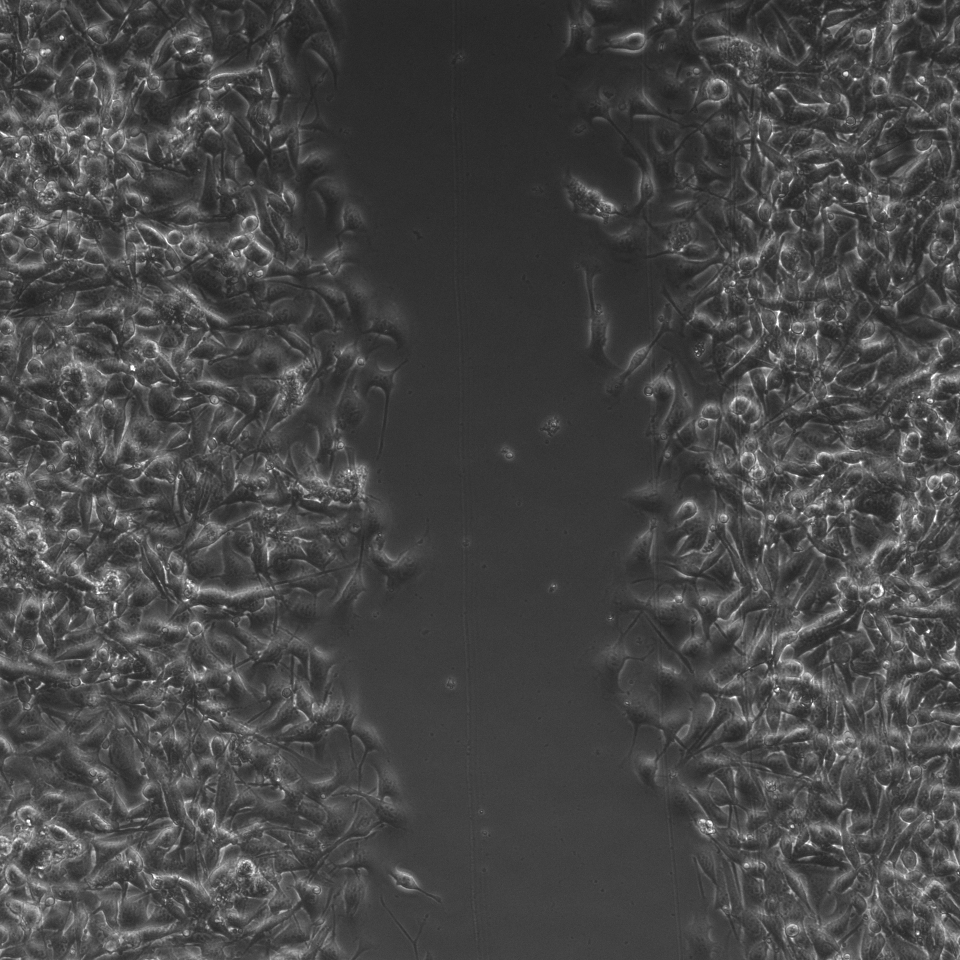

Supplement: Supplementary file 6 — Figure EV Source Data [file 44319_2023_52_MOESM6_ESM.zip › Figure EV/Figure EV4/4F/scramble-16h.tif]

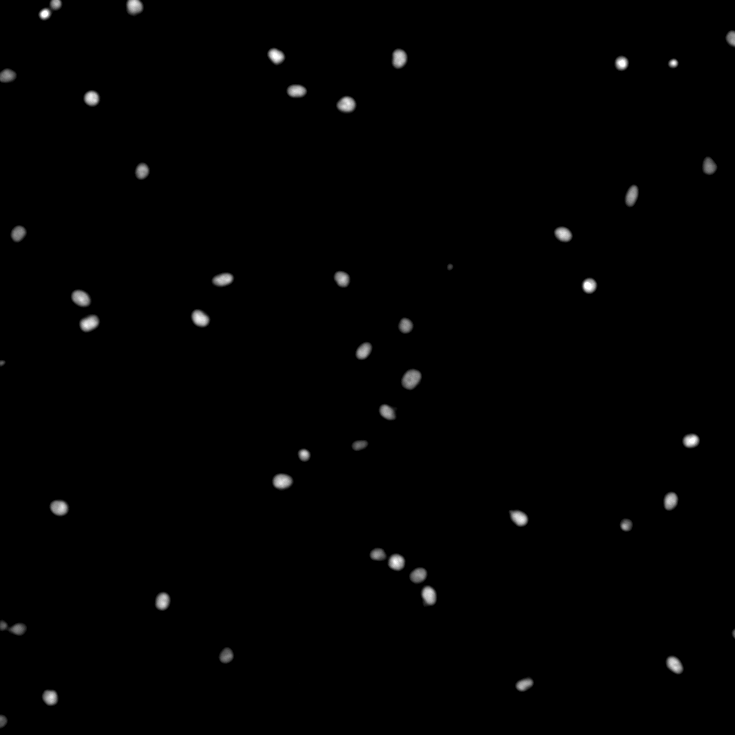

Supplement: Supplementary file 6 — Figure EV Source Data [file 44319_2023_52_MOESM6_ESM.zip › Figure EV/Figure EV5/5B/H2O2+Catalase.tif]

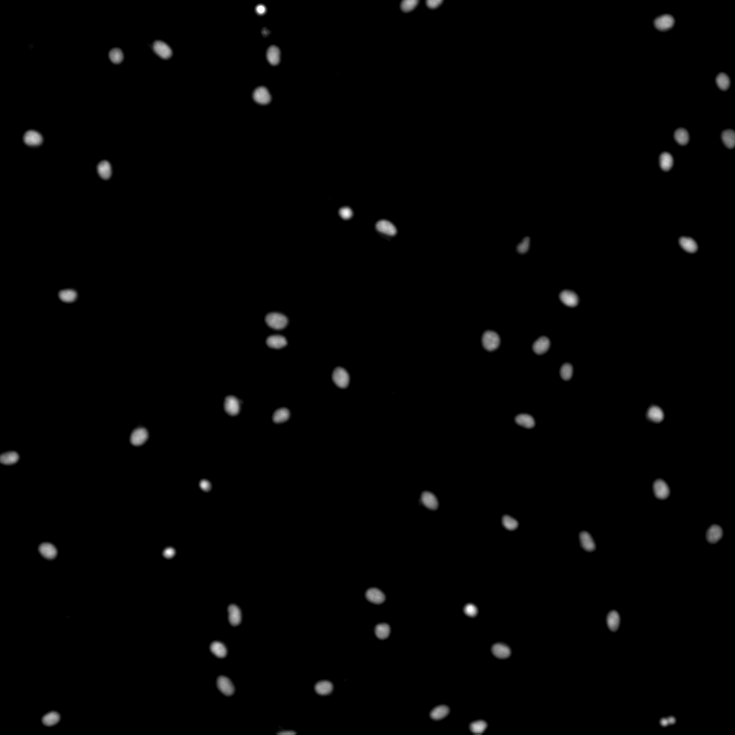

Supplement: Supplementary file 6 — Figure EV Source Data [file 44319_2023_52_MOESM6_ESM.zip › Figure EV/Figure EV5/5B/H2O2.tif]

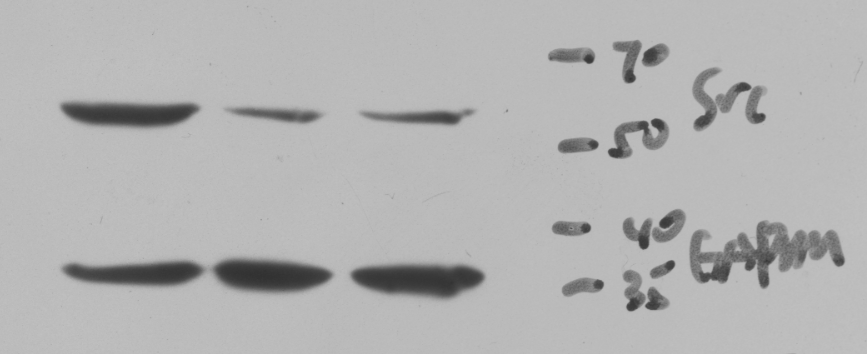

Supplement: Supplementary file 6 — Figure EV Source Data [file 44319_2023_52_MOESM6_ESM.zip › Figure EV/Figure EV5/5E/Src & GAPDH.tif]

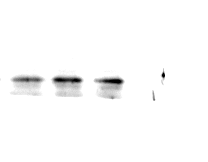

Supplement: Supplementary file 6 — Figure EV Source Data [file 44319_2023_52_MOESM6_ESM.zip › Figure EV/Figure EV5/5F/GAPDH.tif]

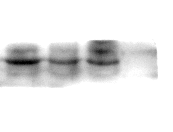

Supplement: Supplementary file 6 — Figure EV Source Data [file 44319_2023_52_MOESM6_ESM.zip › Figure EV/Figure EV5/5F/pSrc.tif]

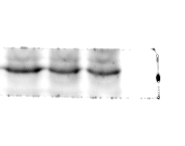

Supplement: Supplementary file 6 — Figure EV Source Data [file 44319_2023_52_MOESM6_ESM.zip › Figure EV/Figure EV5/5F/Total Src.tif]
