# Supplementary material for: Loss of Myo19 increases metastasis by enhancing microenvironmental ROS gradient and chemotaxis
Source: EMBO Rep. 2024 Jan 26;25(3):9. doi: 10.1038/s44319-023-00052-y (PMC10933354; doi:10.1038/s44319-023-00052-y)
Supplement: Supplementary file 8 — Expanded View Figures [file 44319_2023_52_MOESM8_ESM.pdf]

Expanded View Figures

Figure EV1. Cristae sculpting protein Myo19 and Mic60 promoted tumor invasion and metastasis without affecting tumor growth.

(**A**) Myo19 expression in tumors compared to normal tissues in EMBL-EBI (EMBL's European Bioinformatics Institute) database. (**B**) Quantification of Myo19 IHC score and Ki-67 index. Pearson correlation analysis showed no significance. Pearson r = -0.1092, $p = 0.3319$. $N = 81$. (**C**) Representative imaging of Ki-67 IHC staining of breast carcinoma in Myo19 Moderate (M) and Low (L) expression group. Scale bar: 100 µm. (**D**) Quantification of Ki-67 index in Myo19 Low (L), Moderate (M) and High (H) expression groups. Data are shown as mean ± SD. $N_L = 41$, $N_M = 23$, $N_H = 16$. Significance was tested using Fisher's exact test. (**E**) Representative image of 4T1 scramble, Mic60 KD and Myo19 KD solid tumors. (**F**) Quantification of the tumor weight of 4T1 scramble, Mic60 KD and Myo19 KD solid tumors. Data are shown as mean ± SD. $N_{scramble} = 7$, $N_{Mic60\ KD} = 7$, $N_{Myo19\ KD} = 7$. Significance was tested using unpaired Student's *t*-test. (**G**) Quantification of the maximal diameter of 4T1 scramble, Mic60 KD and Myo19 KD solid tumors. Data are shown as mean ± SD. $N_{scramble} = 7$, $N_{Mic60\ KD} = 7$, $N_{Myo19\ KD} = 7$. Significance was tested using unpaired Student's *t*-test. (**H**) Representative electron microscope (EM) images of MDA-MB-231 wild type (WT), Myo19 KO and Mic60 KD cells. Scale bar: 1 µm. Zoomed image scale bar: 300 nm. Source data are available online for this figure.

 © The Author(s)

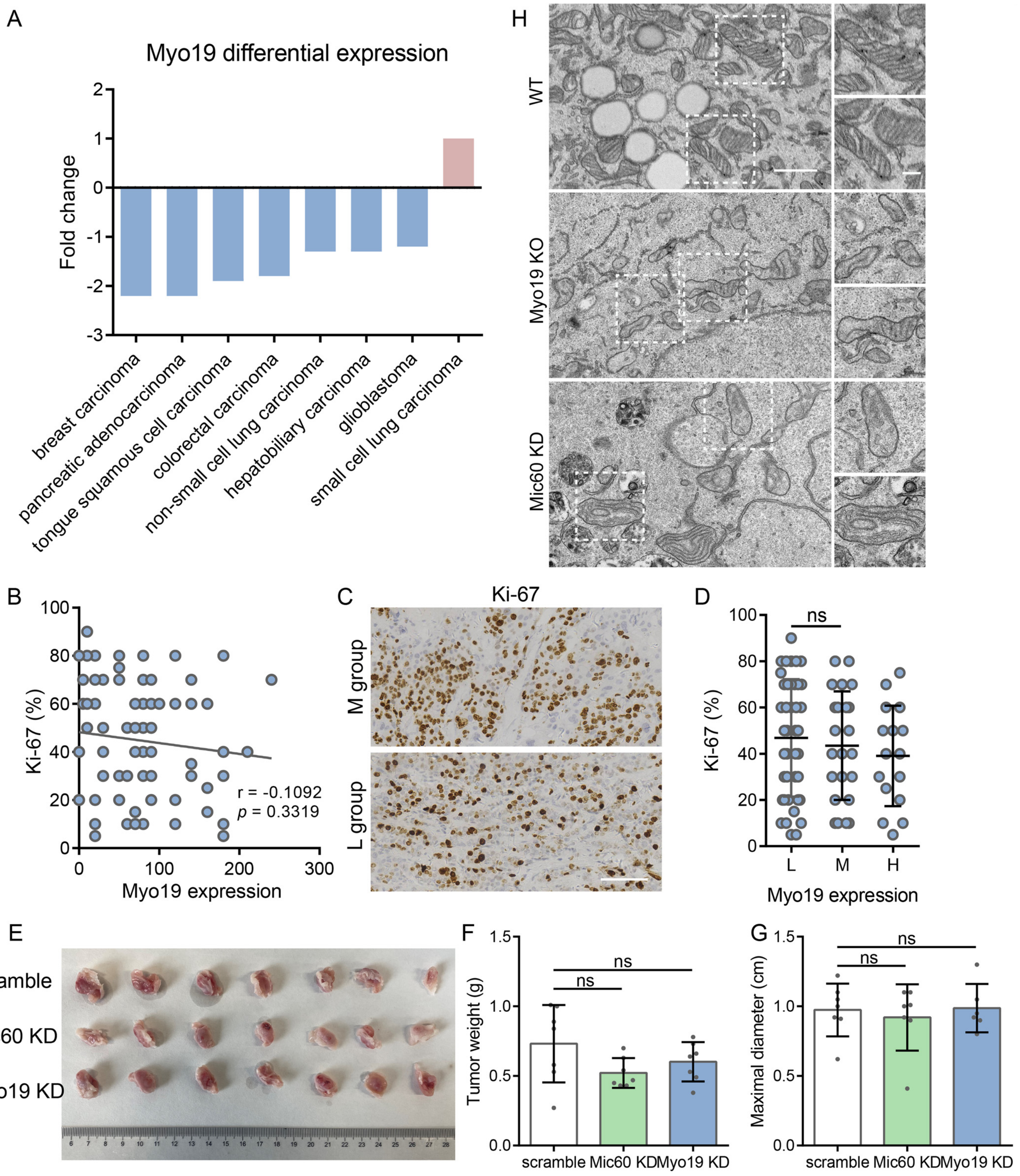
A
Myo19 differential expression
Fold change
2
1
0
-1
-2
-3
breast carcinoma
pancreatic adenocarcinoma
tongue squamous cell carcinoma
colorectal carcinoma
non-small cell lung carcinoma
hepatobiliary carcinoma
glioblastoma
small cell lung carcinoma
H
WT
Myo19 KO
Mic60 KD
B
Ki-67 (%)
r = -0.1092
p = 0.3319
Myo19 expression
C
Ki-67
M group
L group
D
ns
Ki-67 (%)
L
M
H
Myo19 expression
E
scramble
Mic60 KD
Myo19 KD
F
Tumor weight (g)
ns
ns
scramble
Mic60 KD
Myo19 KD
G
Maximal diameter (cm)
ns
ns
scramble
Mic60 KD
Myo19 KD

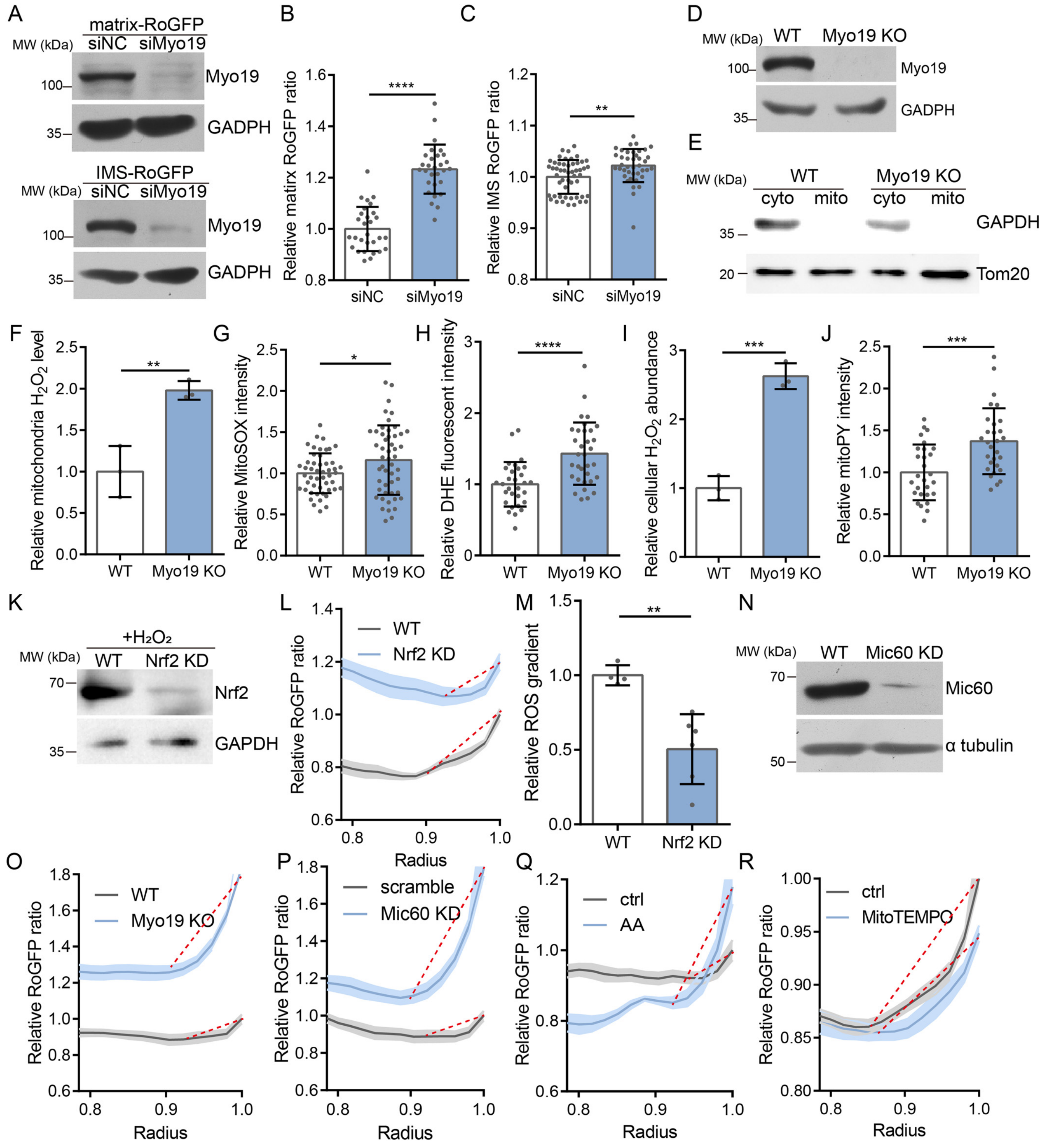
A
matrix-RoGFP
MW (kDa) siNC siMyo19
100
Myo19
35
GADPH
IMS-RoGFP
MW (kDa) siNC siMyo19
100
Myo19
35
GADPH
B
Relative matirx RoGFP ratio
****
siNC siMyo19
C
Relative IMS RoGFP ratio
**
siNC siMyo19
D
MW (kDa) WT Myo19 KO
100
Myo19
35
GADPH
E
MW (kDa) WT Myo19 KO
cyto mito cyto mito
35
GAPDH
20
Tom20
F
Relative mitochondria H2O2 level
**
WT Myo19 KO
G
Relative MitoSOX intensity
*
WT Myo19 KO
H
Relative DHE fluorescent intensity
****
WT Myo19 KO
I
Relative cellular H2O2 abundance
***
WT Myo19 KO
J
Relative mitoPY intensity
***
WT Myo19 KO
K
+H2O2
MW (kDa) WT Nrf2 KD
70
Nrf2
35
GAPDH
L
WT
Nrf2 KD
Relative RoGFP ratio
Radius
M
Relative ROS gradient
**
WT Nrf2 KD
N
MW (kDa) WT Mic60 KD
70
Mic60
50
α tubulin
O
WT
Myo19 KO
Relative RoGFP ratio
Radius
P
scramble
Mic60 KD
Relative RoGFP ratio
Radius
Q
ctrl
AA
Relative RoGFP ratio
Radius
R
ctrl
MitoTEMPO
Relative RoGFP ratio
Radius

 © The Author(s)

Figure EV2. Loss of Myo19 promoted mitochondria ROS production and spheroid ROS gradient.

(**A**) Immunoblotting of mitochondria matrix- and intermembrane space (IMS)-RoGFP expressed MDA-MB-231 cells with siMyo19. GAPDH was used as a loading control. (**B**) Quantification of the RoGFP ratio in matrix-RoGFP expressed cells with siMyo19. Data are shown as mean ± SD. $N_{siNC} = 29$, $N_{siMyo19} = 29$. ****, $p < 0.0001$. Significance was tested using unpaired Student's *t*-test. (**C**) Quantification of the RoGFP ratio in IMS-RoGFP expressed cells with siMyo19. Data are shown as mean ± SD. $N_{siNC} = 51$, $N_{siMyo19} = 40$. **, $p = 0.0020$. Significance was tested using unpaired Student's *t*-test. (**D**) Immunoblotting of WT and Myo19 KO cells. GAPDH was used as a loading control. (**E**) Immunoblotting of isolated mitochondria from MDA-MB-231 WT and Myo19 KO cells. GAPDH was used to indicate cytosolic proteins and Tom 20 was used to indicate mitochondria proteins. (**F**) Quantification of relative H_2O_2 level of isolated mitochondria. Data are shown as mean ± SD. $N_{WT} = 3$, $N_{Myo19\ KO} = 3$. **, $p = 0.0067$. Significance was tested using unpaired Student's *t*-test. (**G**) Quantification of relative MitoSOX intensity in MDA-MB-231 WT and Myo19 KO cells. Cells were treated with 500 nM MitoSOX for 30 minutes. Data are shown as mean ± SD. $N_{WT} = 49$, $N_{Myo19\ KO} = 50$. *, $p = 0.0233$. Significance was tested using unpaired Student's *t*-test. (**H**) Quantification of relative DHE intensity of MDA-MB-231 WT and Myo19 KO cells. Cells were treated with 10 μM DHE for 20 minutes. Data are shown as mean ± SD. $N_{WT} = 30$, $N_{Myo19\ KO} = 33$. ****$p < 0.0001$. Significance was tested using unpaired Student's *t*-test. (**I**) Quantification of relative H_2O_2 level in WT and Myo19 KO cell lysis. Data are shown as mean ± SD. $N_{WT} = 3$, $N_{Myo19\ KO} = 3$. ***$p = 0.0004$. Significance was tested using unpaired Student's *t*-test. (**J**) Quantification of relative mitoPY intensity in MDA-MB-231 WT and Myo19 KO cells. Cells were treated with 20 μM mitoPY for 3 h. Data are shown as mean ± SD. $N_{WT} = 28$, $N_{Myo19\ KO} = 29$. ***$p = 0.0003$. Significance was tested using unpaired Student's *t*-test. (**K**) Immunoblotting of MDA-MB-231 WT and Nrf2 KD cells treated with 100 μM H_2O_2 for 1 h. GAPDH was used as a loading control. (**L**) Quantification of the relative RoGFP ratio along radius in WT and Nrf2 KD spheroids. Red dash line indicates ROS gradient. Data are shown as mean ± SEM. $N_{WT} = 4$, $N_{Nrf2\ KD} = 6$. (**M**) Quantification of the relative ROS gradient in WT and Nrf2 KD spheroids. Data are shown as mean ± SD. $N_{WT} = 4$, $N_{Nrf2\ KD} = 6$. **$p = 0.0037$. Significance was tested using unpaired Student's *t*-test. (**N**) Immunoblotting of WT and Mic60 KD cells. α tubulin was used as a loading control. (**O**) Quantification of the relative RoGFP ratio along radius in WT and Myo19 KO spheroids. Red dash line indicates ROS gradient. Data are shown as mean ± SEM. $N_{WT} = 6$, $N_{Myo19\ KO} = 6$. (**P**) Quantification of the relative RoGFP ratio along radius in WT and Mic60 KD spheroids. Red dash line indicates ROS gradient. Data are shown as mean ± SEM. $N_{WT} = 6$, $N_{Mic60\ KD} = 5$. (**Q**) Quantification of the relative RoGFP ratio along radius in spheroids treated with 1 μM Antimycin A (AA) during the whole formation process. Red dash line indicates ROS gradient. Data are shown as mean ± SEM. $N_{ctrl} = 6$, $N_{AA} = 4$. (**R**) Quantification of the relative RoGFP ratio along radius in spheroids treated with 20 μM MitoTEMPO for 3 hours. Red dash line indicates ROS gradient. Data are shown as mean ± SEM. $N_{ctrl} = 6$, $N_{MitoTEMPO} = 6$. Source data are available online for this figure.

© The Author(s)

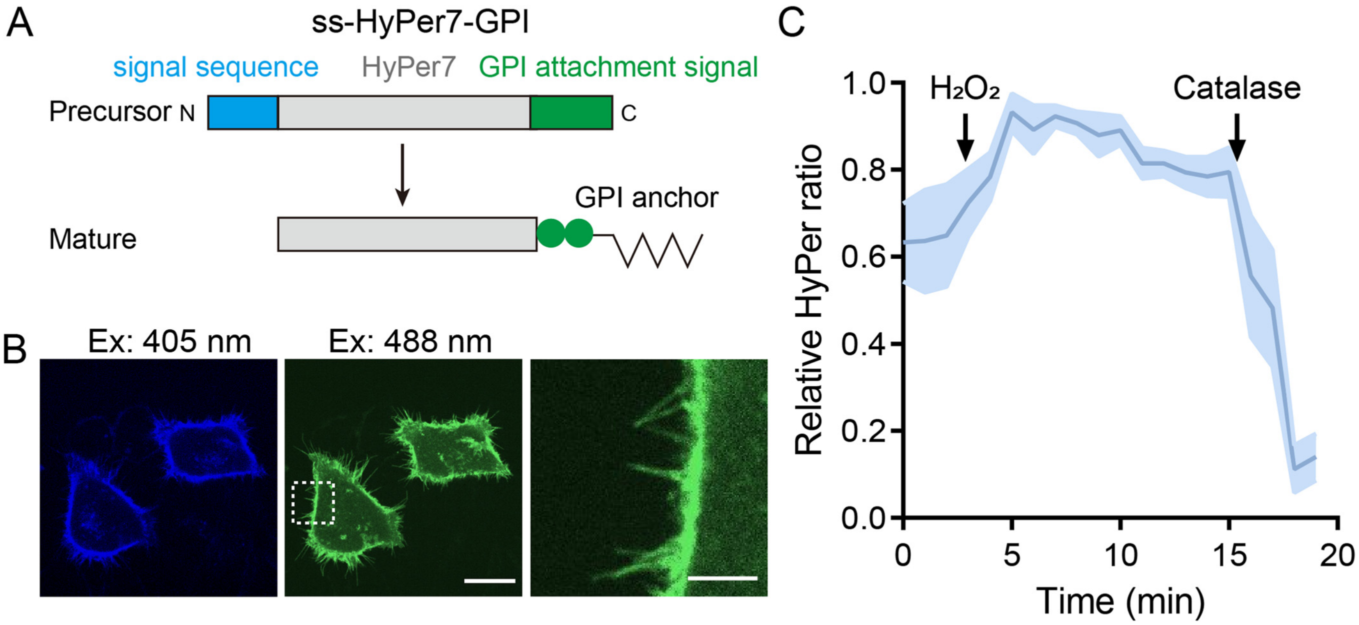

Figure EV3. ss-HyPer7-GPI probe indicated extracellular H_2O_2 level.

(**A**) Schematic diagram showing the construct of ss-HyPer7-GPI probe. The HyPer7 sensor was fused with N-terminal endoplasmic reticulum (ER)-targeting signal sequence (ss), and C-terminal GPI-attachment signal. In the mature probe, these two sequences of the precursor probe were removed and GPI was attached to HyPer7. (**B**) Localization of the HyPer7 fluorescence in MDA-MB-231 cells expressing ss-HyPer7-GPI. Scale bar: 20 μm. Scale bar of the cropped images: 10 μm. (**C**) Quantification of the relative HyPer ratio in MDA-MB-231 cells expressing ss-HyPer7-GPI. 150 μM H_2O_2 and 2000 U/mL catalase were added in the culture medium at the indicated time. $N = 7$. Source data are available online for this figure.

 © The Author(s)

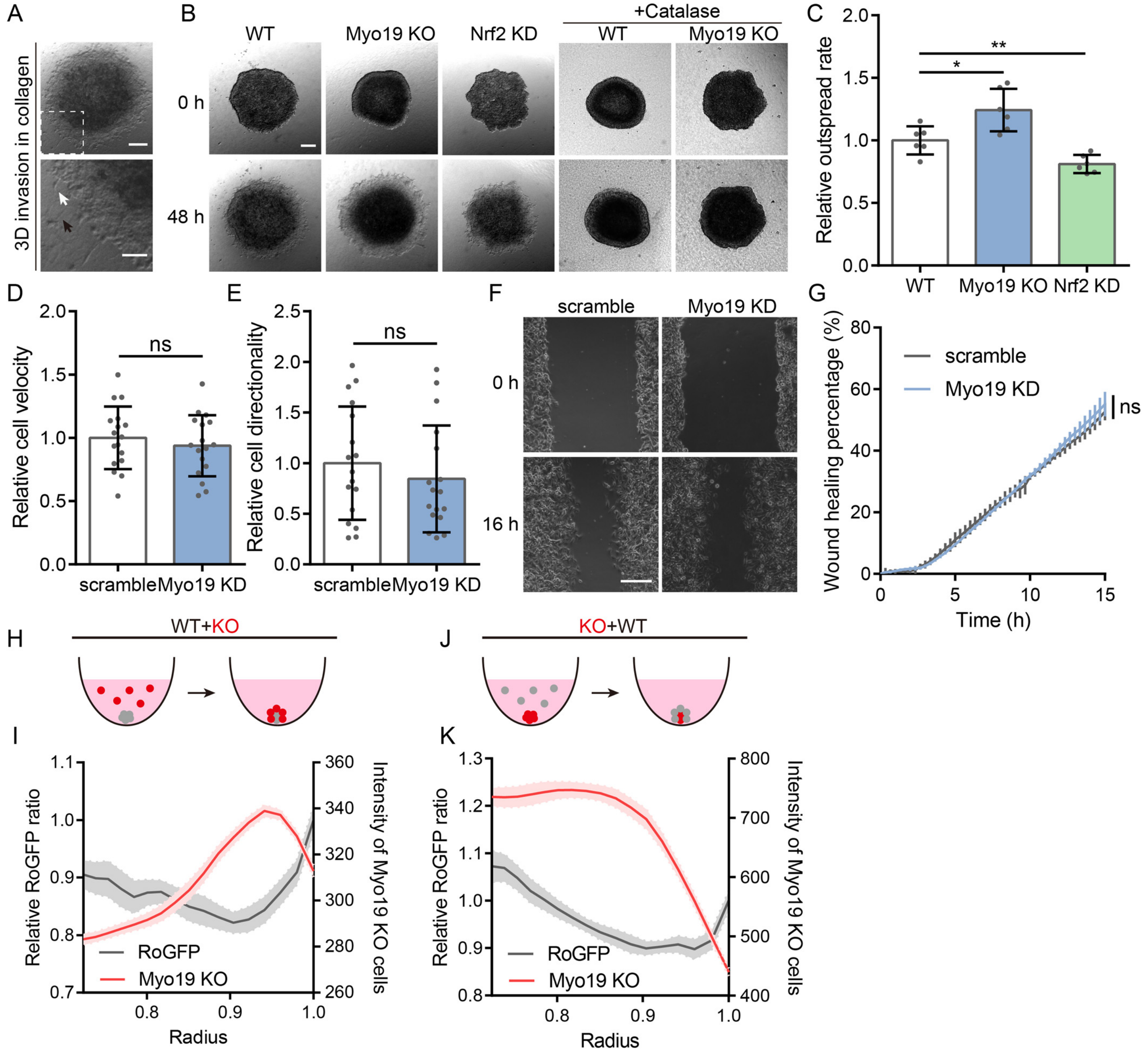

Figure EV4. Loss of Myo19 promoted 3D spheroid invasion but not migratory ability.

(**A**) Up: representative imaging of MDA-MB-231 spheroid invasion in 3.5 mg/mL rat tail collagen. Scale bar: 200 μm. Down: the cropped image in the white square. White arrow indicates the invading cell. Black arrow indicates the collagen fiber. Scale bar: 100 μm. (**B**) Representative images of spheroid invasion in 3.5 mg/mL rat tail collagen. 5000 U/mL catalase was added during 48 hours of invasion. Scale bar: 200 μm. (**C**) Quantification of relative outspread rate in WT, Myo19 KO and Nrf2 KD spheroids during 48 hours of invasion. Data are shown as mean ± SD. $N_{WT} = 6$, $N_{Myo19\ KO} = 6$, $N_{Nrf2\ KD} = 6$. *$p = 0.0154$. **$p = 0.0061$. Significance was tested using unpaired Student's *t*-test. (**D**) Quantification of the velocity of B16-F10 cell random motion. Data are shown as mean ± SD. $N_{scramble} = 17$, $N_{Myo19\ KD} = 17$. Significance was tested using unpaired Student's *t*-test. (**E**) Quantification of the directionality of B16-F10 cell random motion. Data are shown as mean ± SD. $N_{scramble} = 17$, $N_{Myo19\ KD} = 17$. Significance was tested using unpaired Student's *t*-test. (**F**) Representative imaging of wound scratch assay of B16-F10 scramble and Myo19 KD cells. Scale bar: 200 μm. (**G**) Quantification of percentage of wound healing within 16 hours. Data are shown as mean ± SD. $N_{scramble} = 5$. $N_{Myo19\ KD} = 5$. Significance was tested using unpaired Student's *t*-test. (**H**) Schematic diagram showing the generation of WT + KO layered spheroid. Myo19 KO cells were pre-stained with CellTracker™ Red CMTPX. 9500 WT cells were used to pre-form WT spheroid, followed by addition of 500 Myo19 KO (red) cells as the outer shell. (**I**) Quantification of the relative RoGFP ratio and the fluorescent intensity of cell dye-stained Myo19 KO cells in WT + KO spheroids. Myo19 KO cells were pre-stained with CellTracker™ Red CMTPX. Data are shown as mean ± SEM. $N = 6$. (**J**) Schematic diagram showing the generation of KO + WT layered spheroid. Myo19 KO cells were pre-stained with CellTracker™ Red CMTPX. 9500 Myo19 KO (red) cells were used to pre-form Myo19 KO spheroid, followed by addition of 500 WT cells as the outer shell. (**K**) Quantification of the relative RoGFP ratio and the fluorescent intensity of cell dye-stained Myo19 KO cells in KO + WT spheroids. Myo19 KO cells were pre-stained with CellTracker™ Red CMTPX. Data are shown as mean ± SEM. $N = 6$. Source data are available online for this figure.

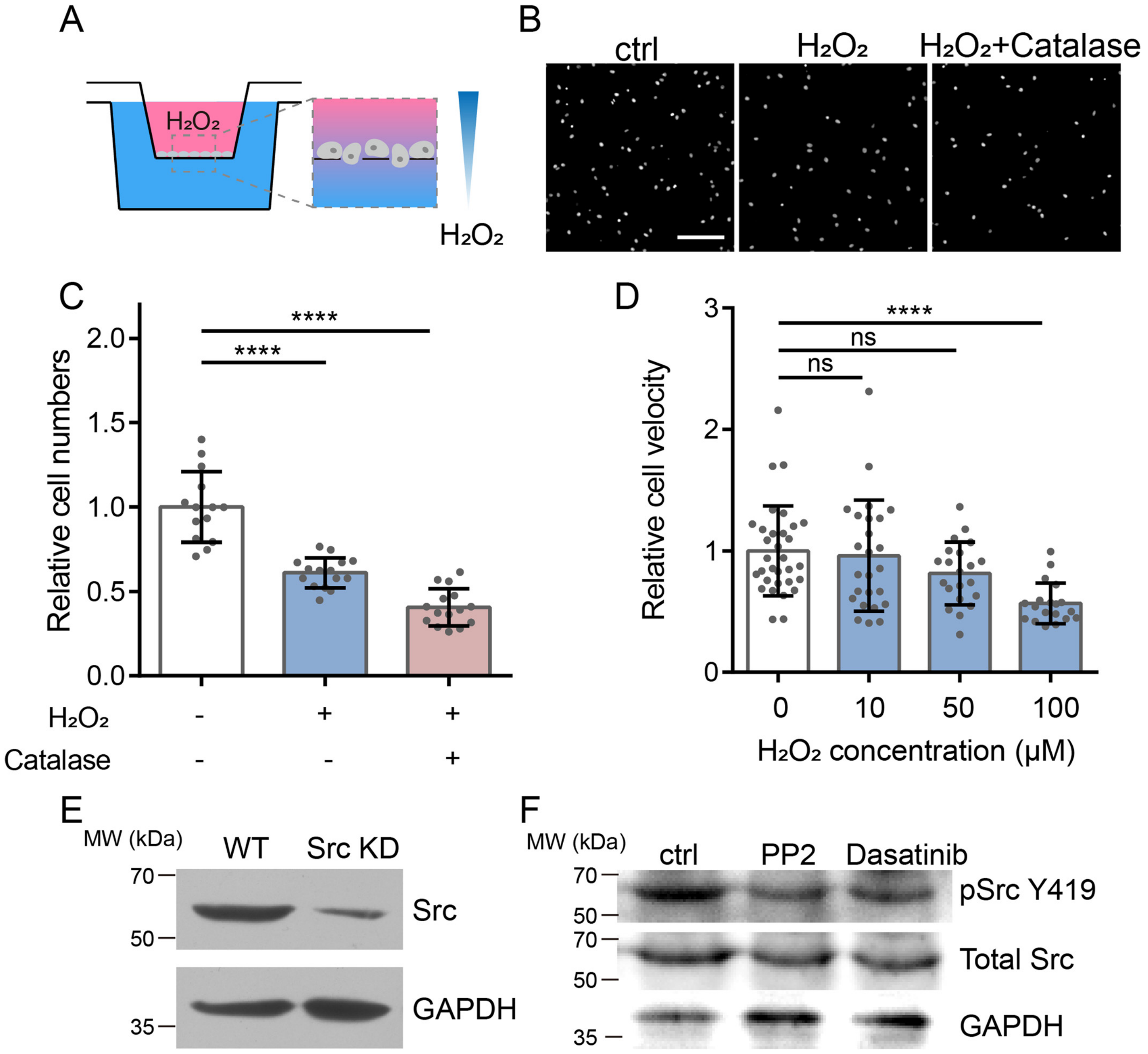

Figure EV5. Direct H_2O_2 stimulation inhibited the velocity of cell migration.

(**A**) Schematic diagram showing the reverse H_2O_2 chemotaxis assay using the Boyden chamber transwell assay. MDA-MB-231 cells and 100 μM H_2O_2 were added in the upper chamber. 1000 U/mL catalase was also used to scavenge H_2O_2 in the H_2O_2+Catalase group. After 12 hours of migration, cells were stained with 2.5 μg/mL Hoechst for 20 min and counted. (**B**) Representative imaging of cells in the lower chamber. Scale bar: 200 μm. (**C**) Quantification of cell numbers in the lower chambers. Data are shown as mean ± SD. $N_{ctrl} = 14$, $N_{H2O2} = 15$, $N_{H2O2+catalase} = 15$. ****$p < 0.0001$. Significance was tested using unpaired Student's *t*-test. (**D**) Quantification of the relative velocity of B16-F10 random cell motion. Cells were treated with H_2O_2 of indicated concentration. Images were captured for 10 hours at the interval of 20 minutes. Data are shown as mean ± SD. $N_{0\ \mu M} = 32$, $N_{10\ \mu M} = 25$, $N_{50\ \mu M} = 21$, $N_{100\ \mu M} = 19$, ****$p < 0.0001$. Significance was tested using unpaired Student's *t*-test. (**E**) Immunoblotting of MDA-MB-231 WT and Src KD cells. GAPDH was used as a loading control. (**F**) Immunoblotting of MDA-MB-231 cells treated with 20 μM PP2 or 10 μM Dasatinib for 12 h. GAPDH was used as a loading control. Source data are available online for this figure.

 © The Author(s)
